# Supplementary material for: Dual origin of relapses in retinoic-acid resistant acute promyelocytic leukemia
Source: Nat Commun. 2018 May 24;9:2047. doi: 10.1038/s41467-018-04384-5 (PMC5967331; doi:10.1038/s41467-018-04384-5)
Supplement: Supplementary file 1 — Supplementary Information [file 41467_2018_4384_MOESM1_ESM.pdf]

Supplementary Informations :

Dual origin of relapses in retinoic-acid resistant acute promyelocytic leukemia
Lehmann-Che et al,

Supplementary Figure 1: copy number profiles of all patients with chromosomal alterations.

Patient P16

Diagnosis

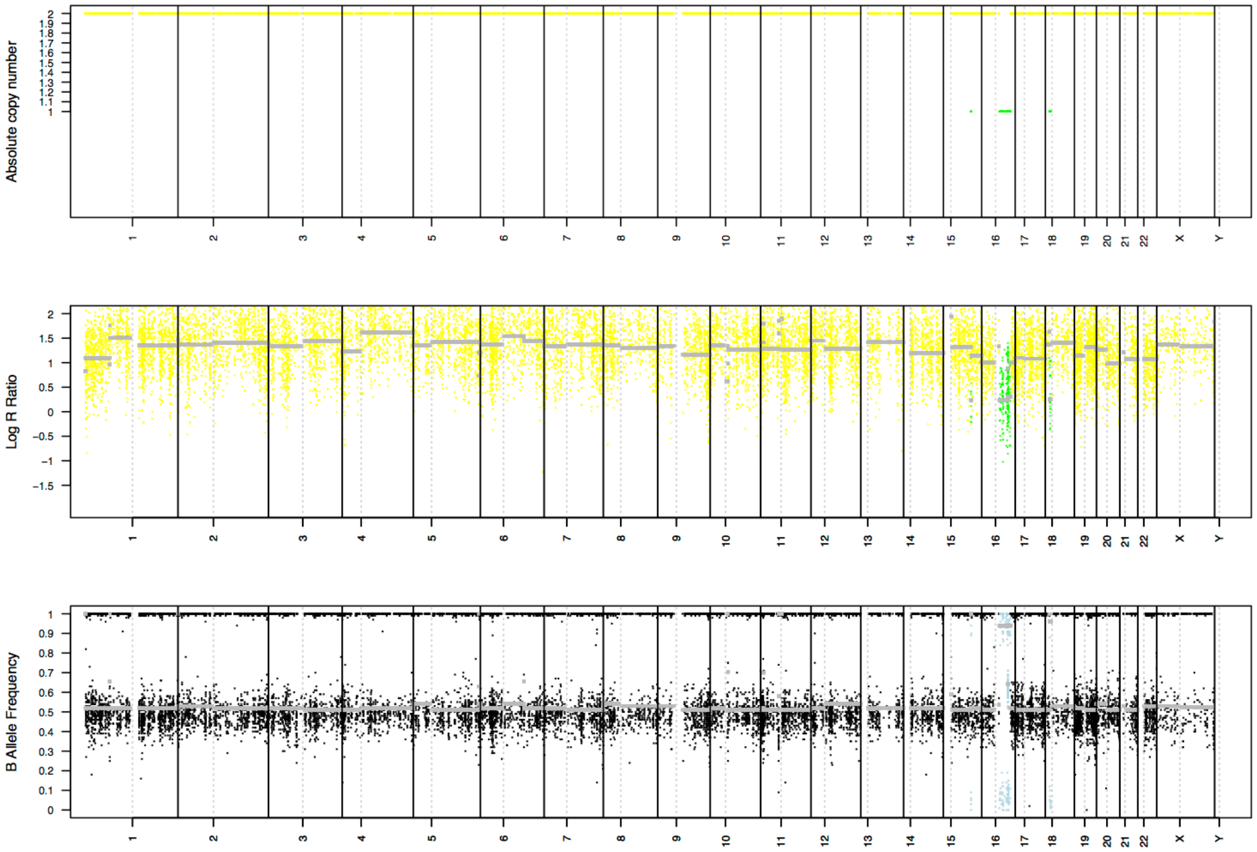

Relapse

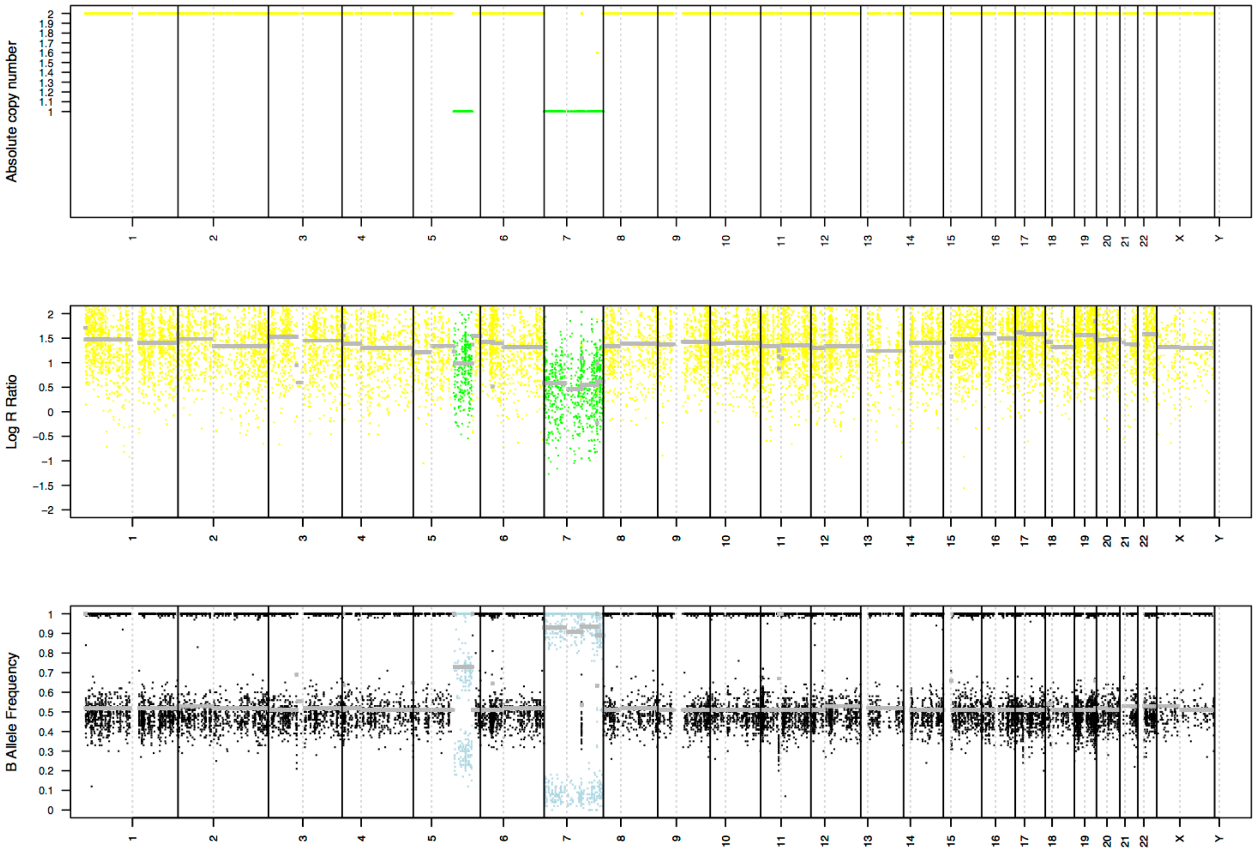

Color code: yellow=normal copy-number, green = deletion, red=gain, lightblue=loss of heterozygosity.

Patient P2

Diagnosis

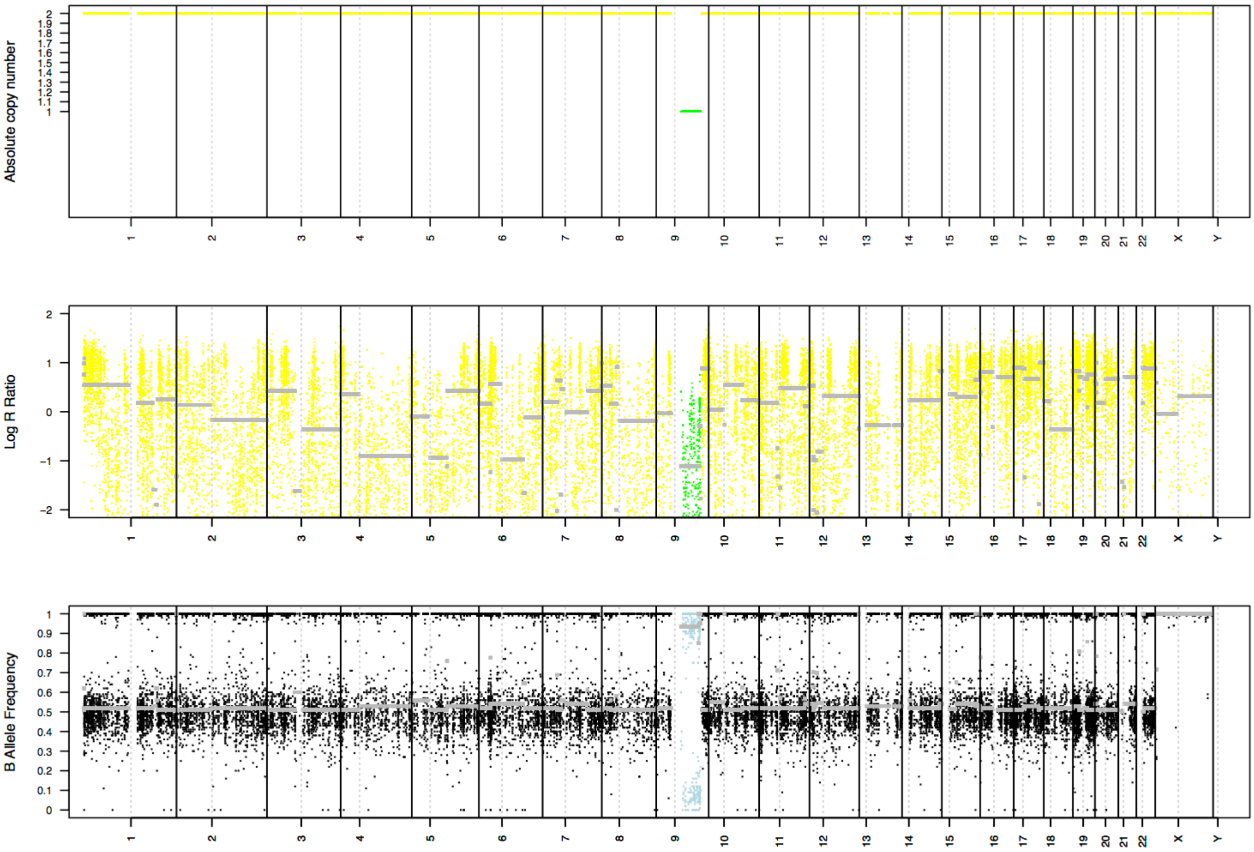

Relapse

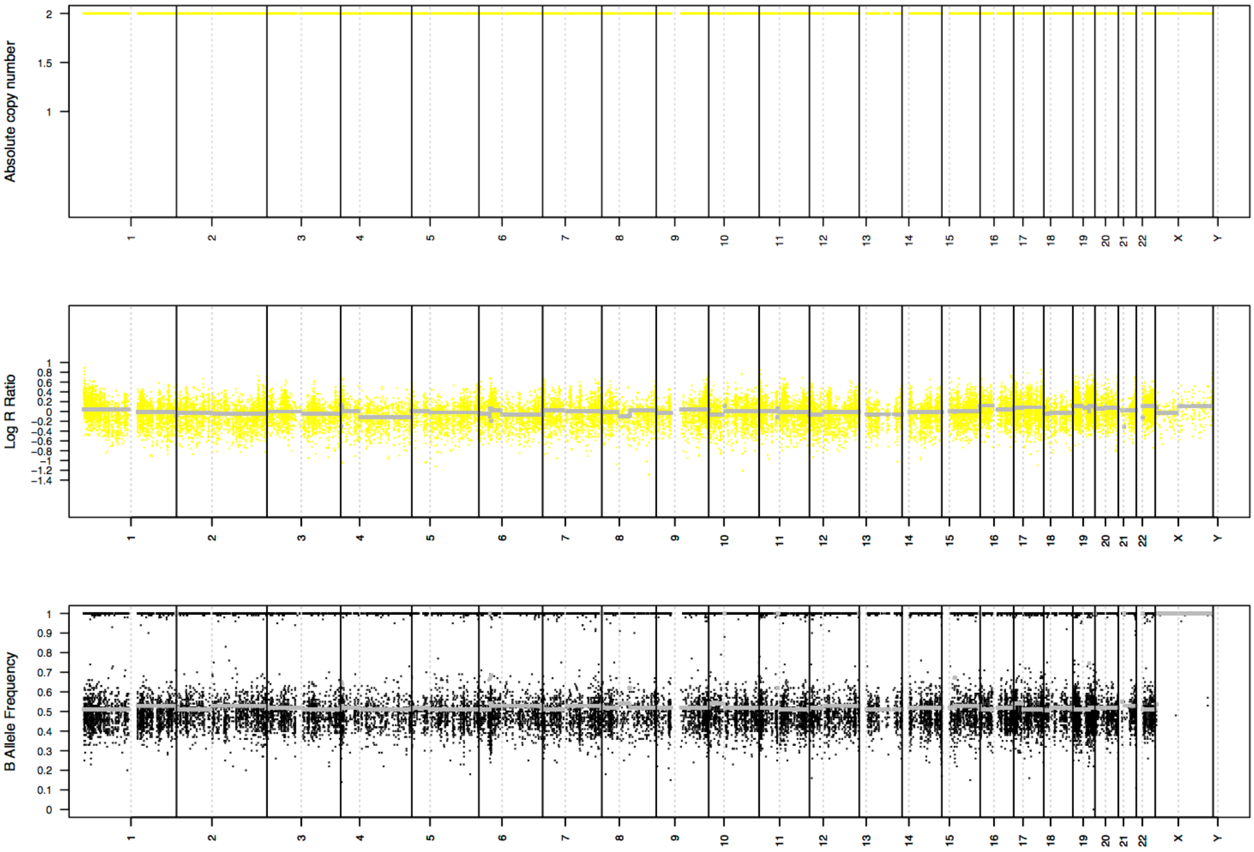

Color code: yellow=normal copy-number, green = deletion, red=gain, lightblue=loss of heterozygosity.

Patient P21

Diagnosis

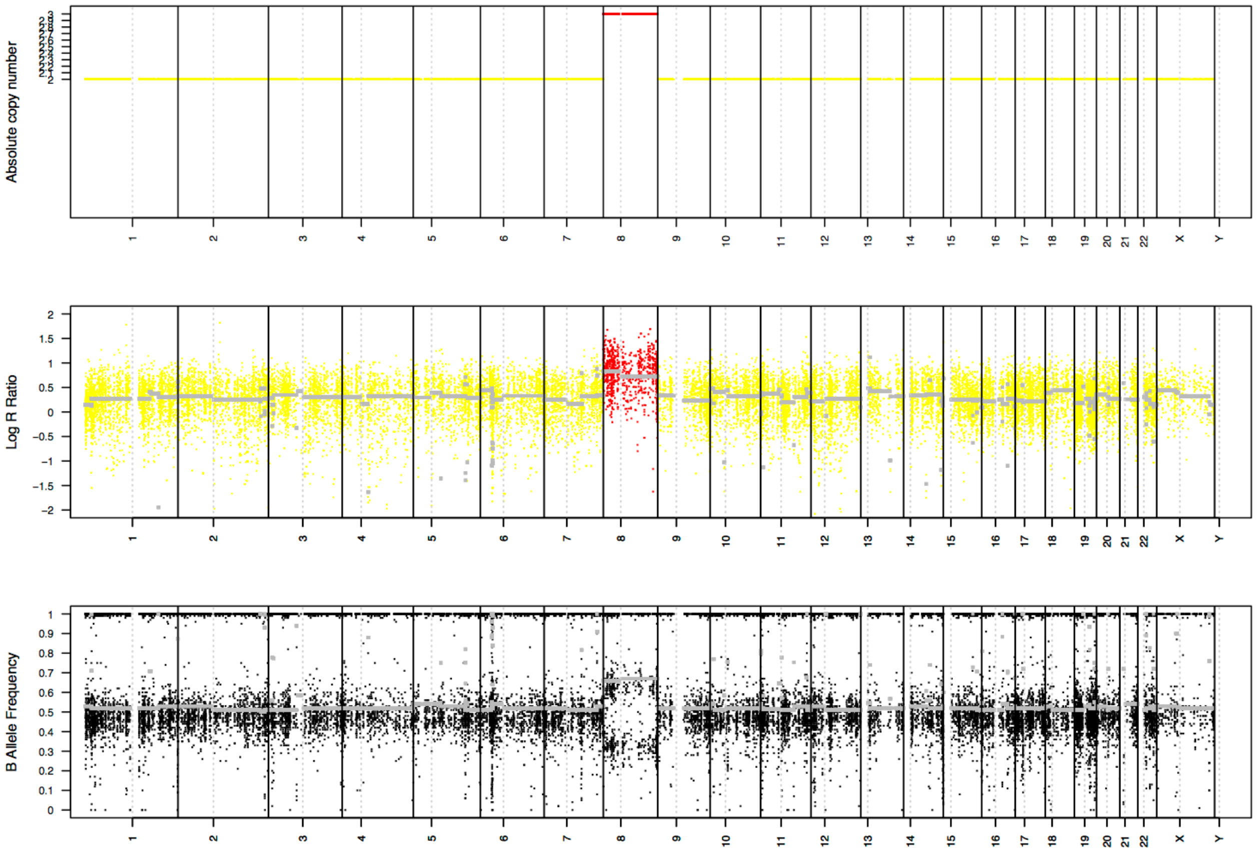

Relapse

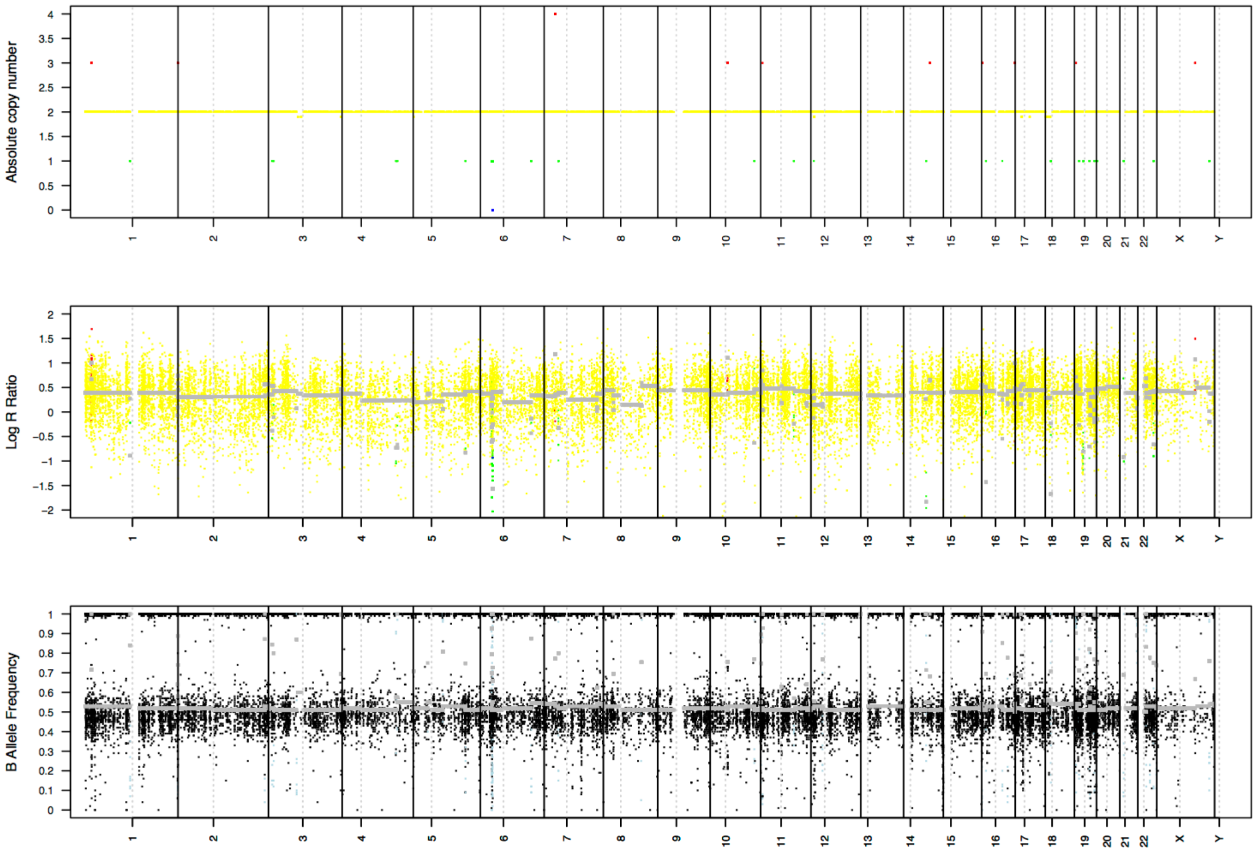

Color code: yellow=normal copy-number, green = deletion, red=gain, lightblue=loss of heterozygosity.

Patient P22

Diagnosis

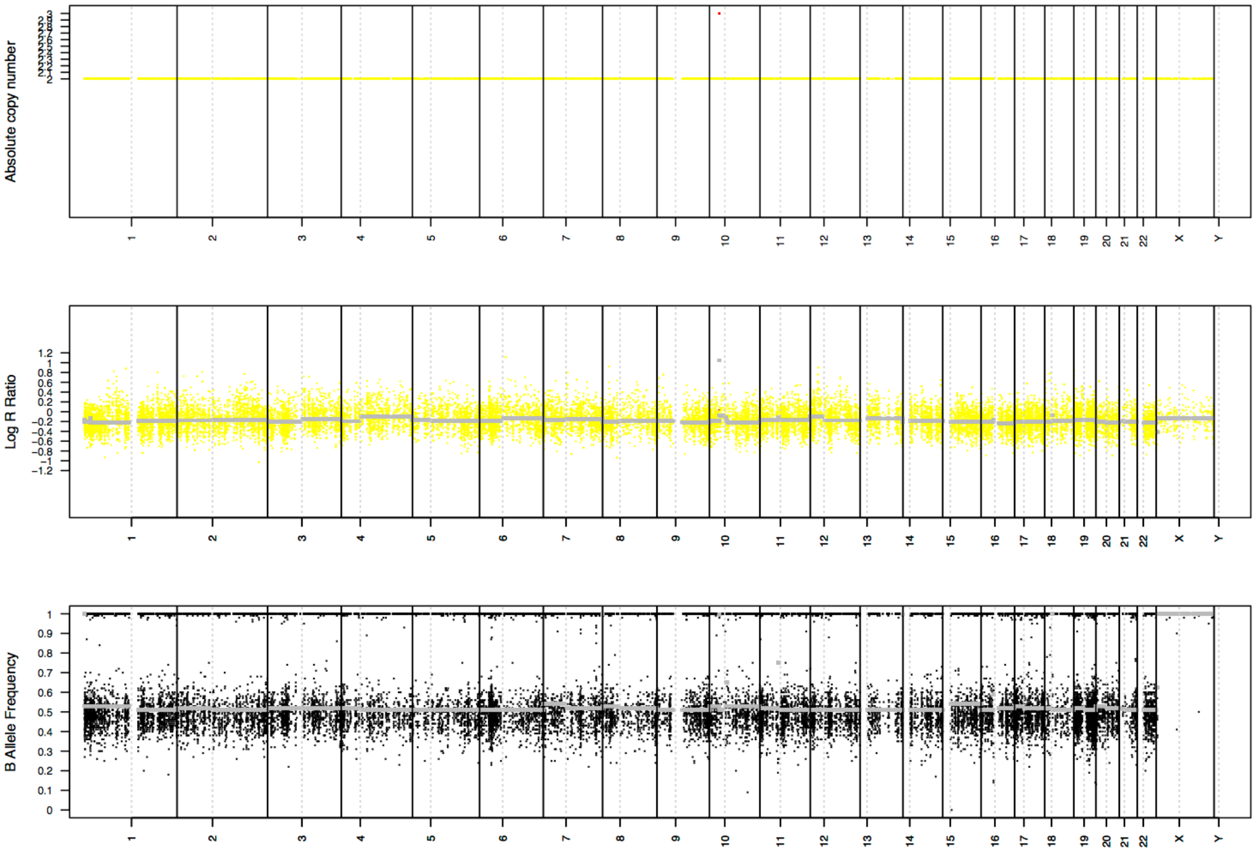

Relapse

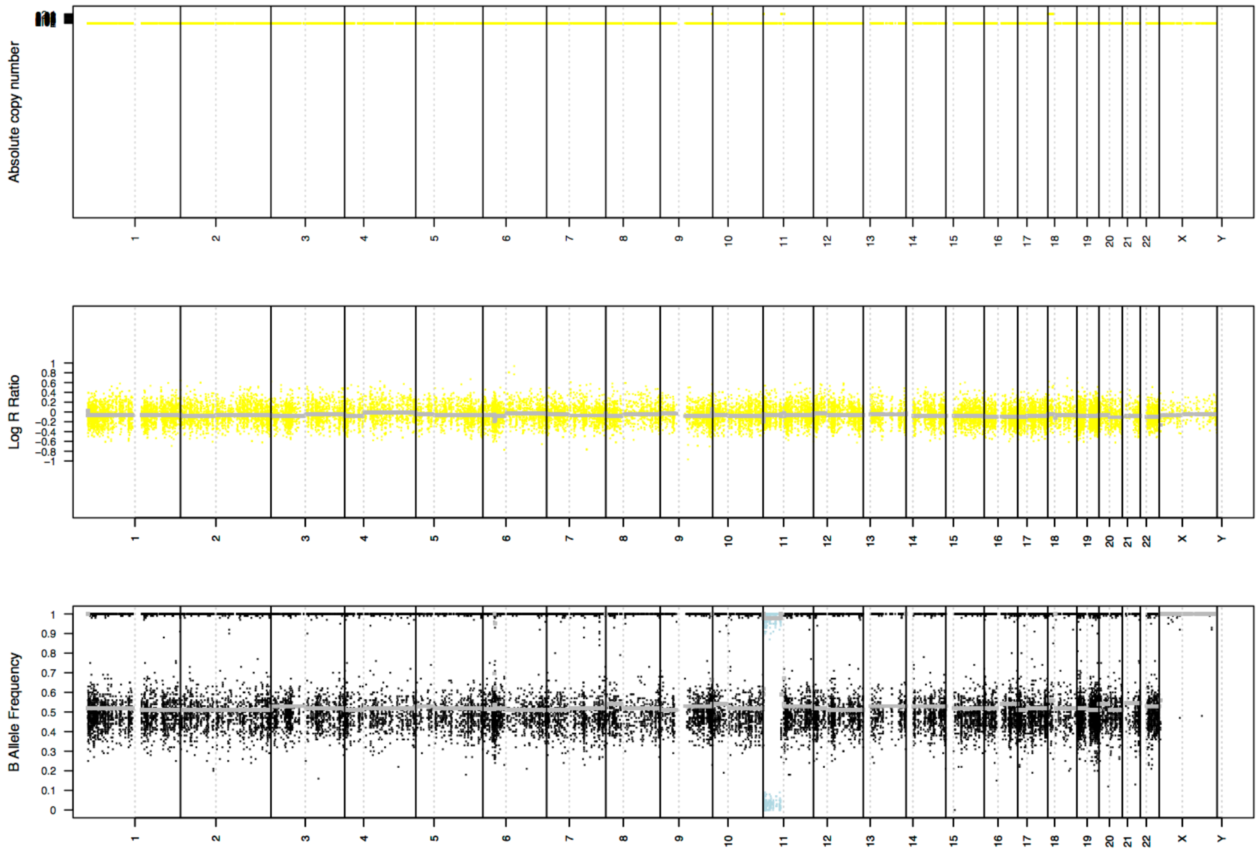

Color code: yellow=normal copy-number, green = deletion, red=gain, lightblue=loss of heterozygosity.

Patient P26

Diagnosis

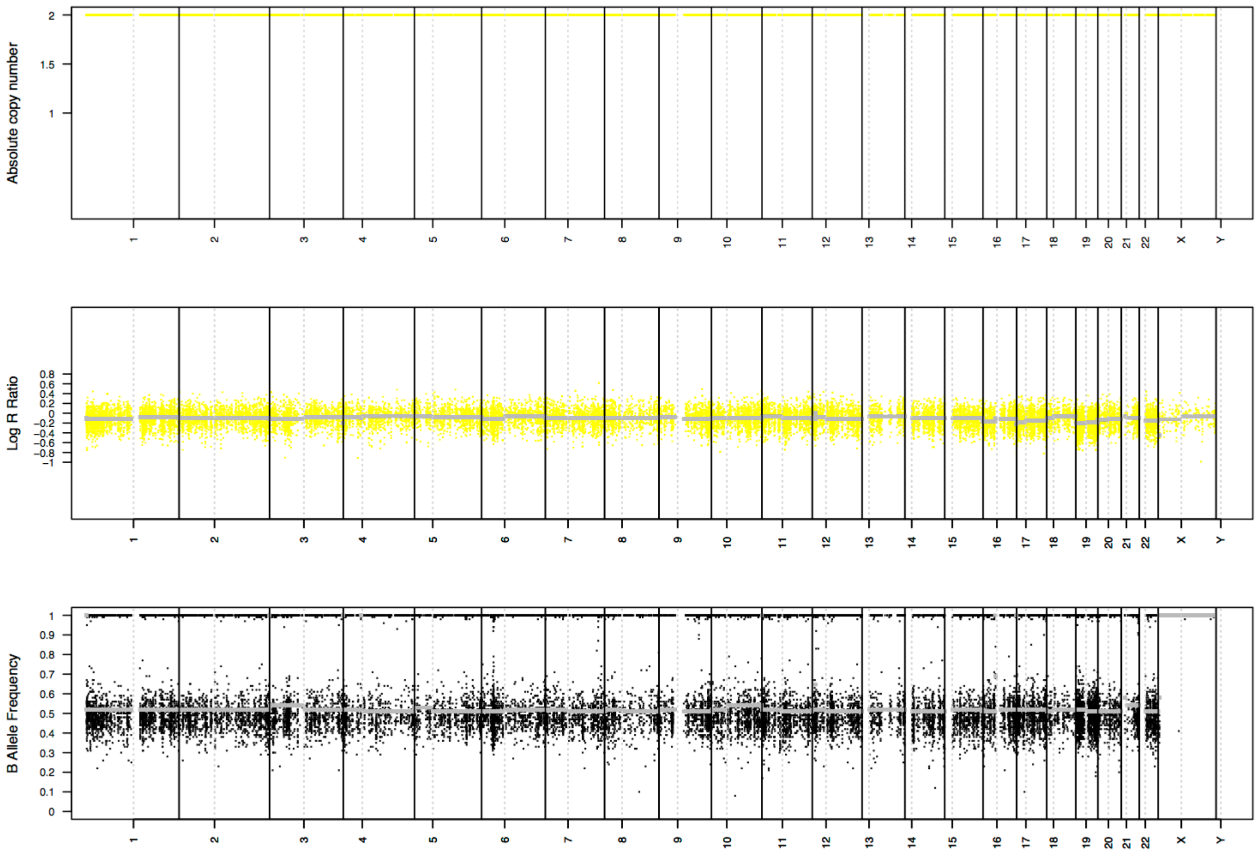

Relapse

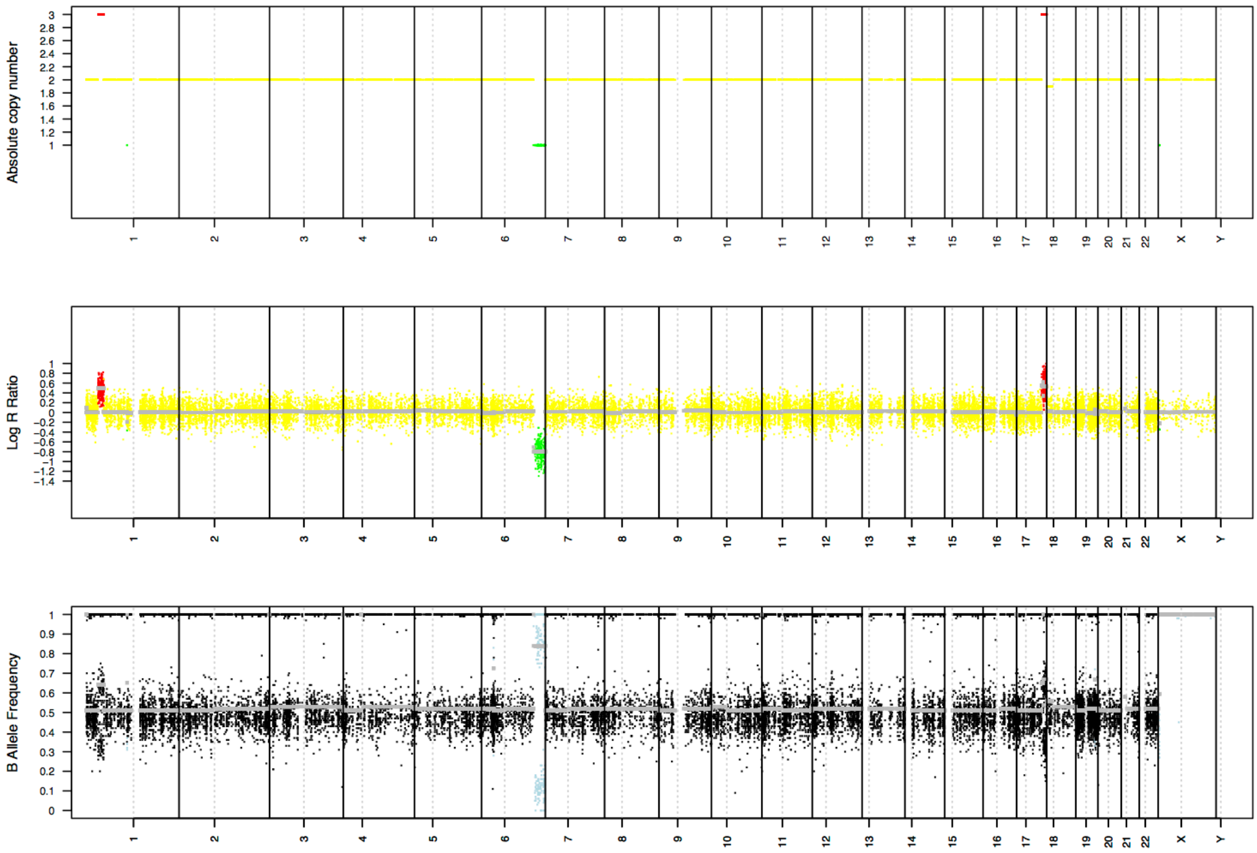

Color code: yellow=normal copy-number, green = deletion, red=gain, lightblue=loss of heterozygosity.

Patient P29

Diagnosis

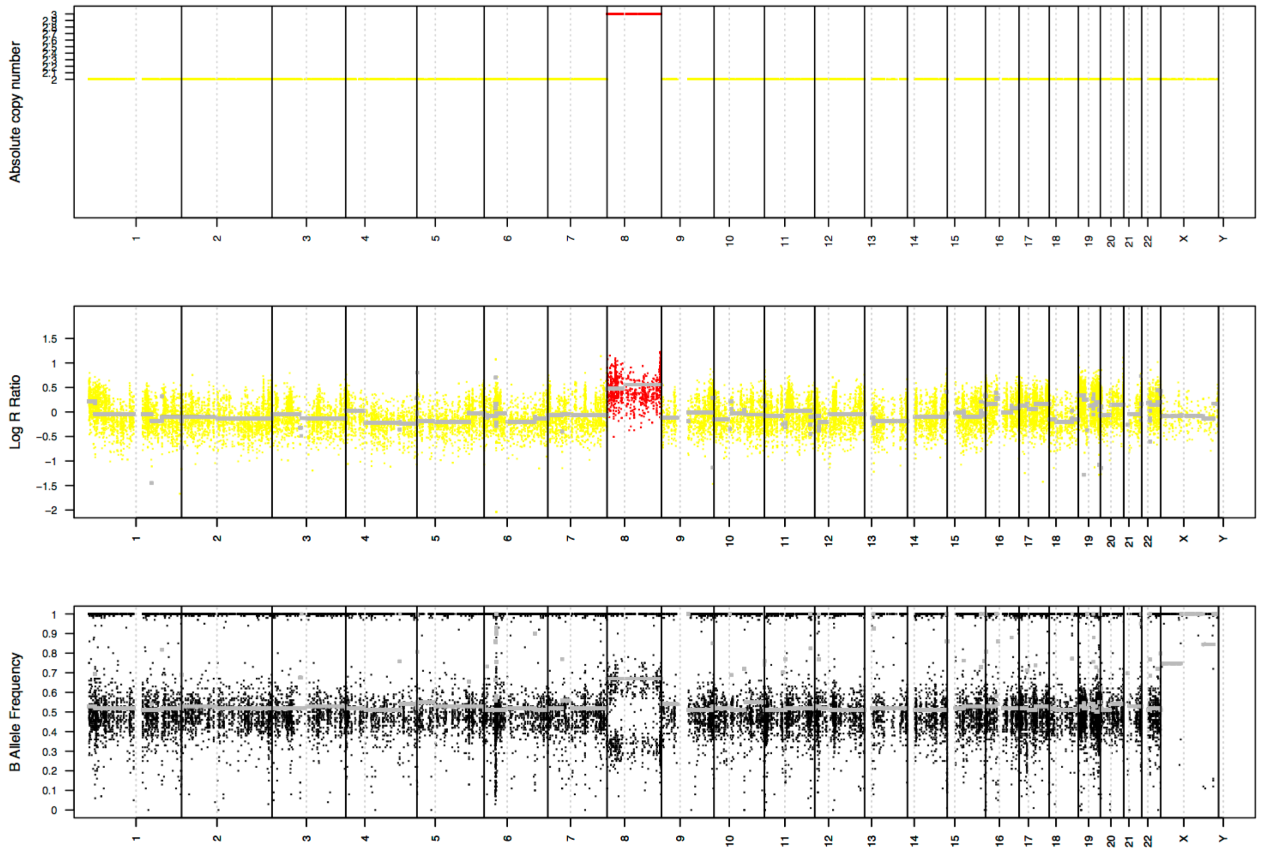

Relapse

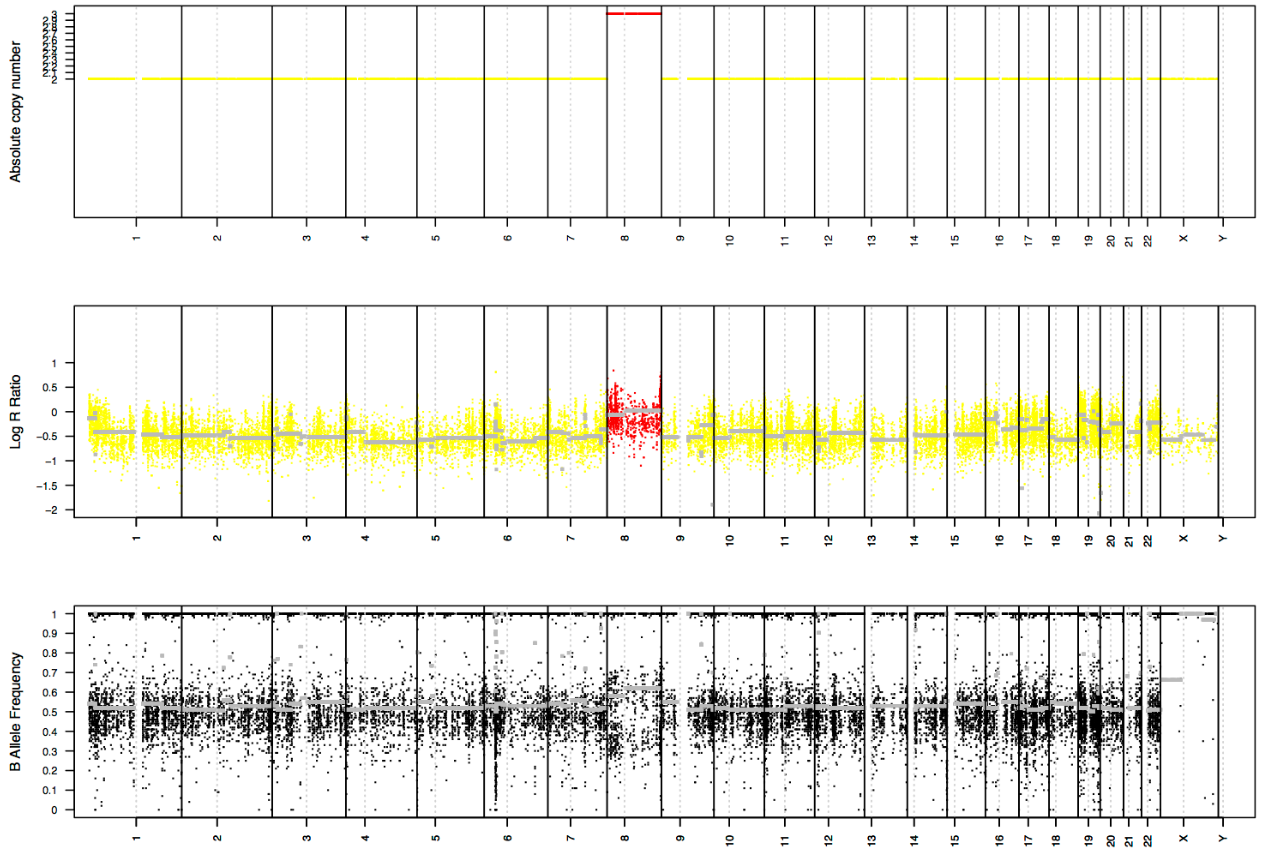

Color code: yellow=normal copy-number, green = deletion, red=gain, lightblue=loss of heterozygosity.

Patient P30

Diagnosis

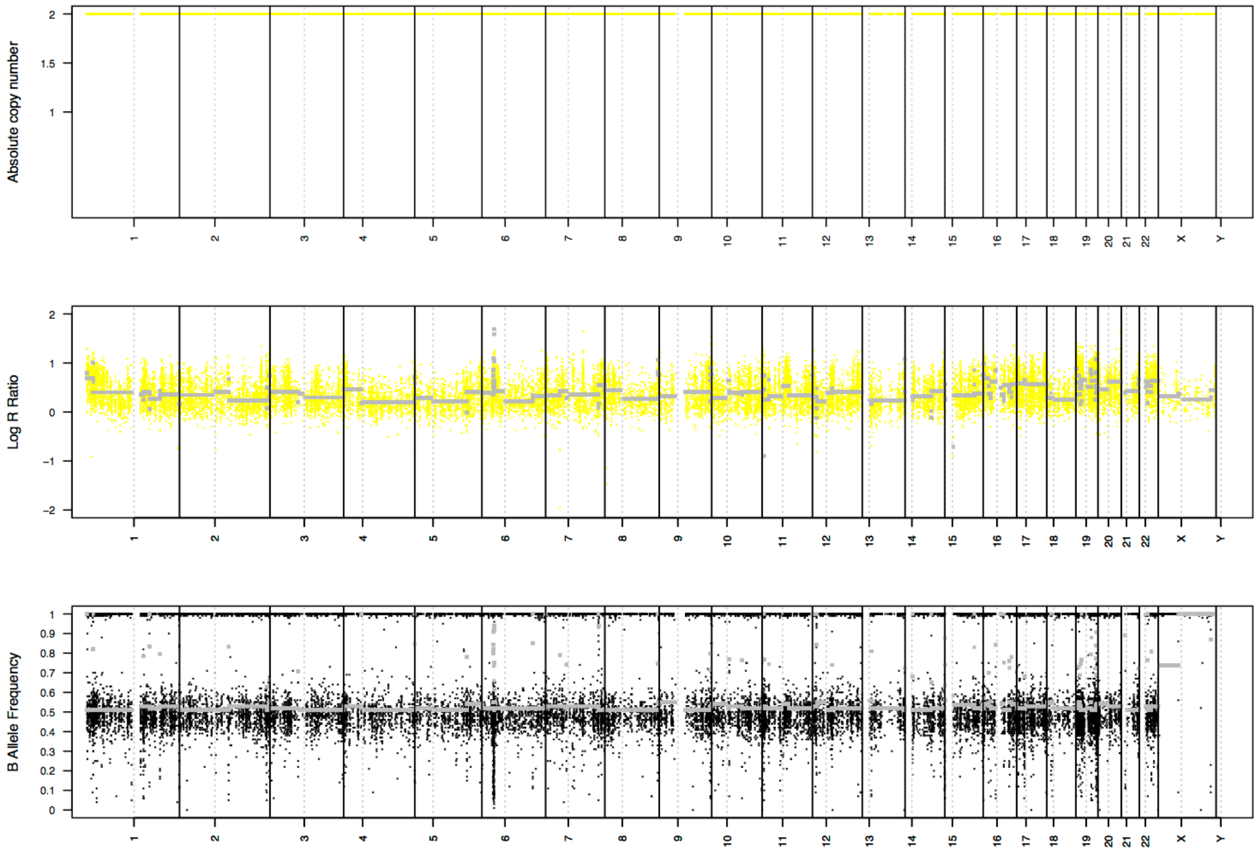

Relapse

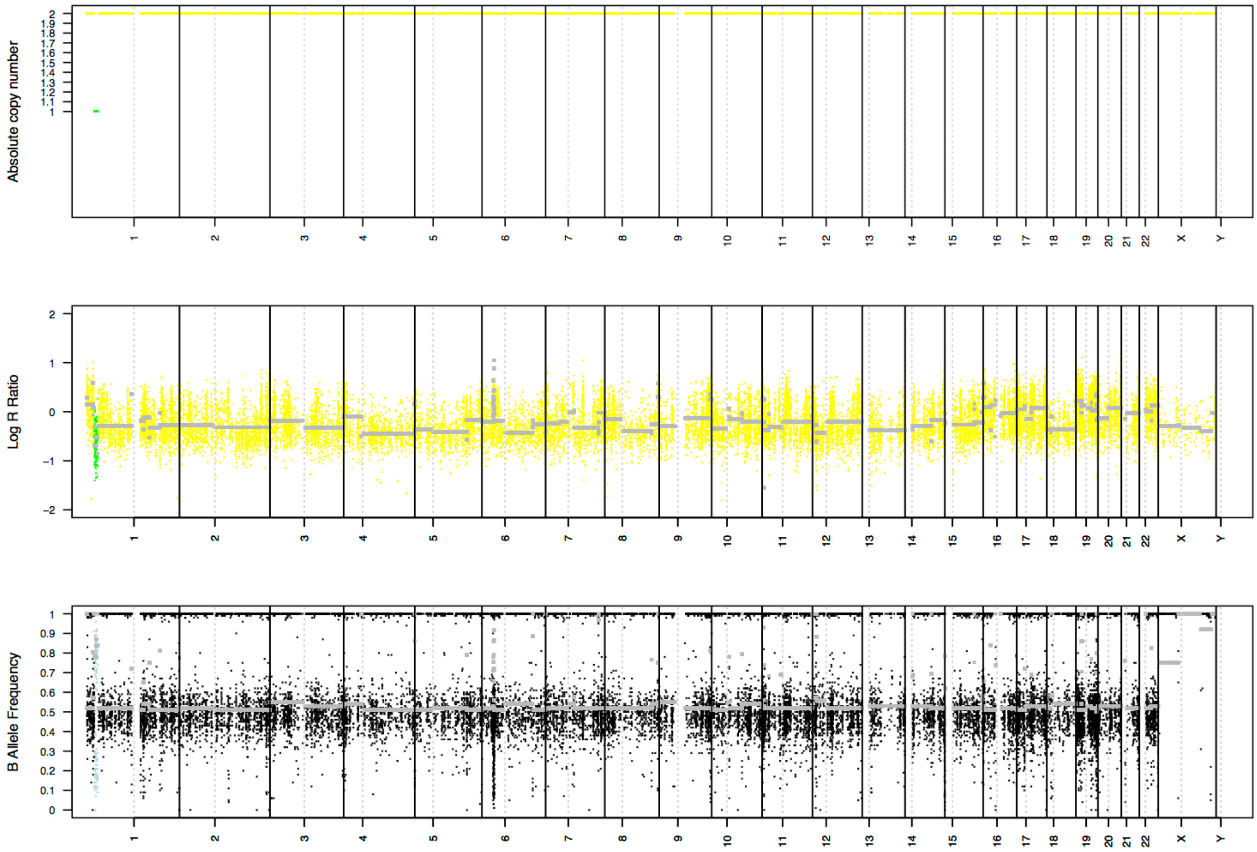

Color code: yellow=normal copy-number, green = deletion, red=gain, lightblue=loss of heterozygosity.

Patient P31

Diagnosis

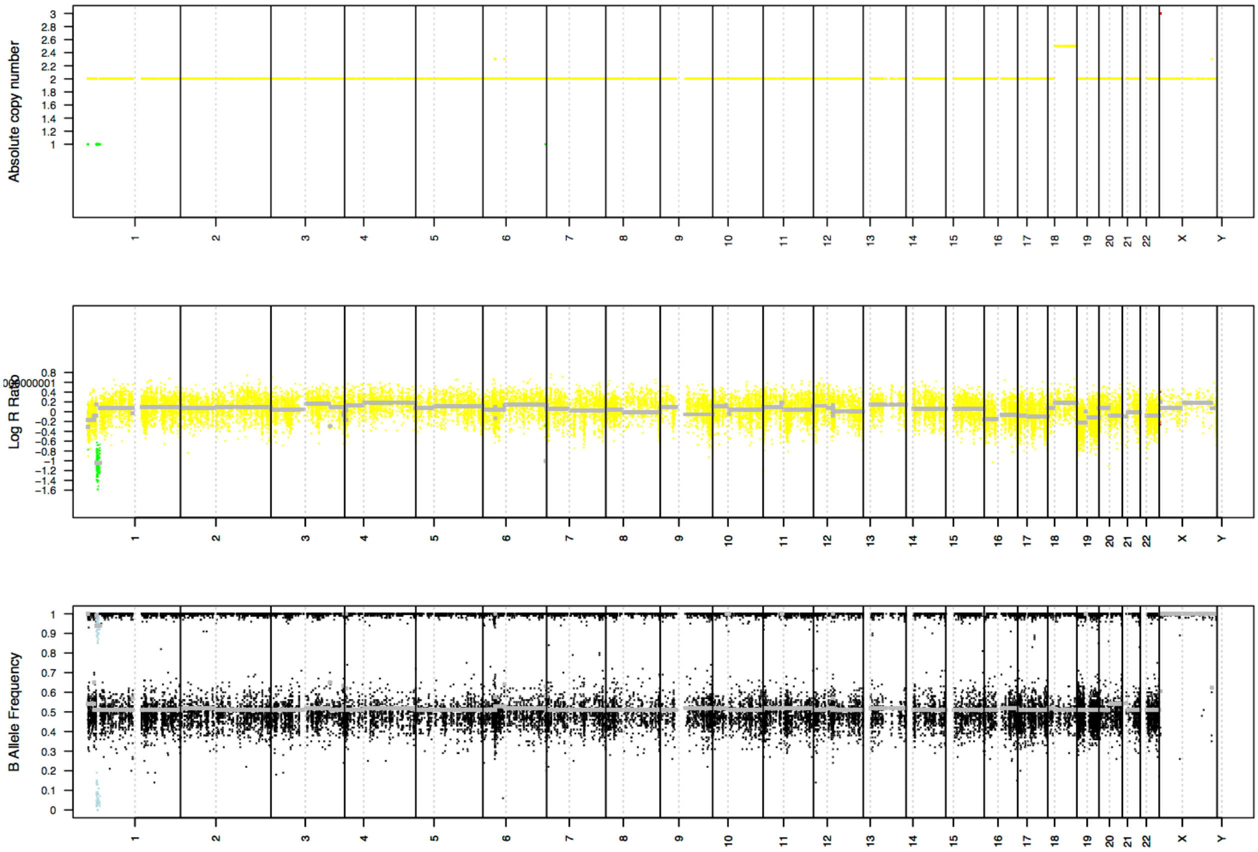

Relapse

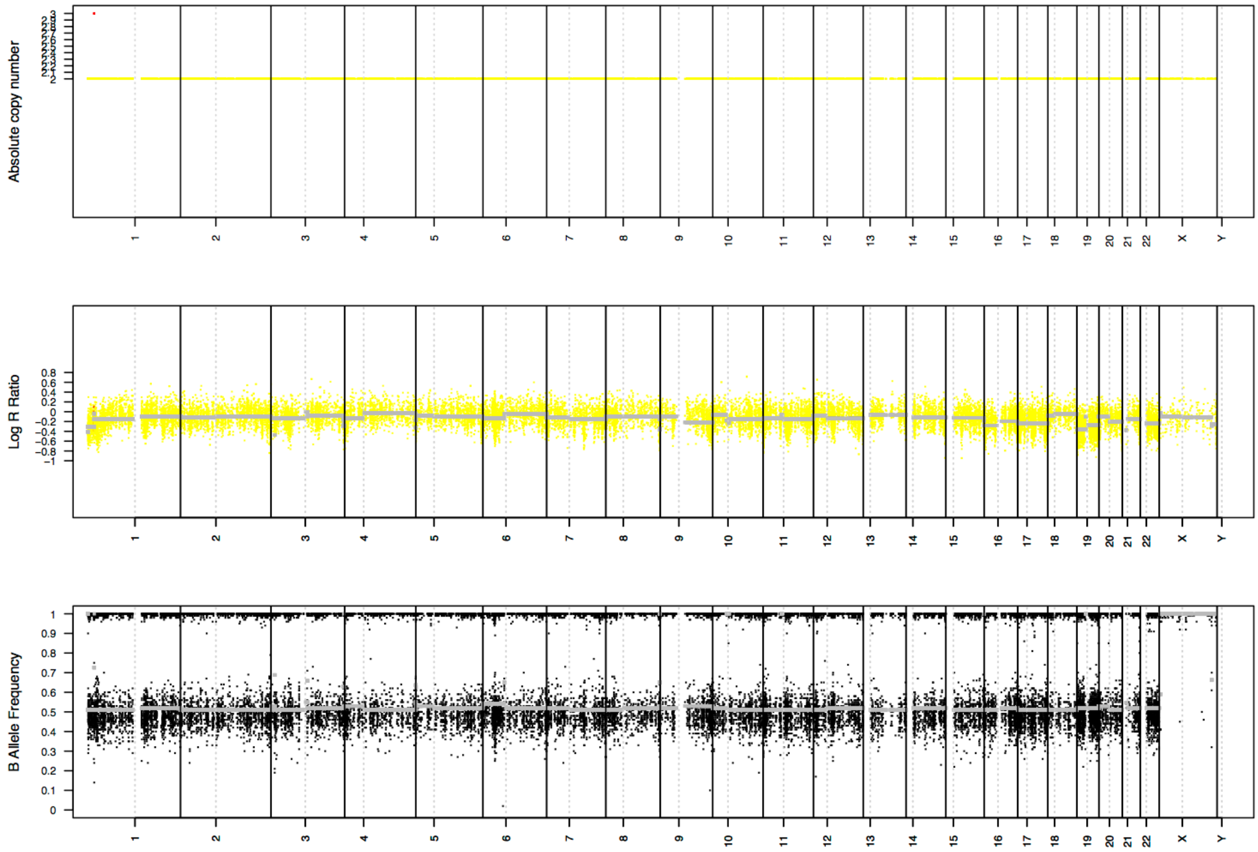

Color code: yellow=normal copy-number, green = deletion, red=gain, lightblue=loss of heterozygosity.

Patient P32

Diagnosis

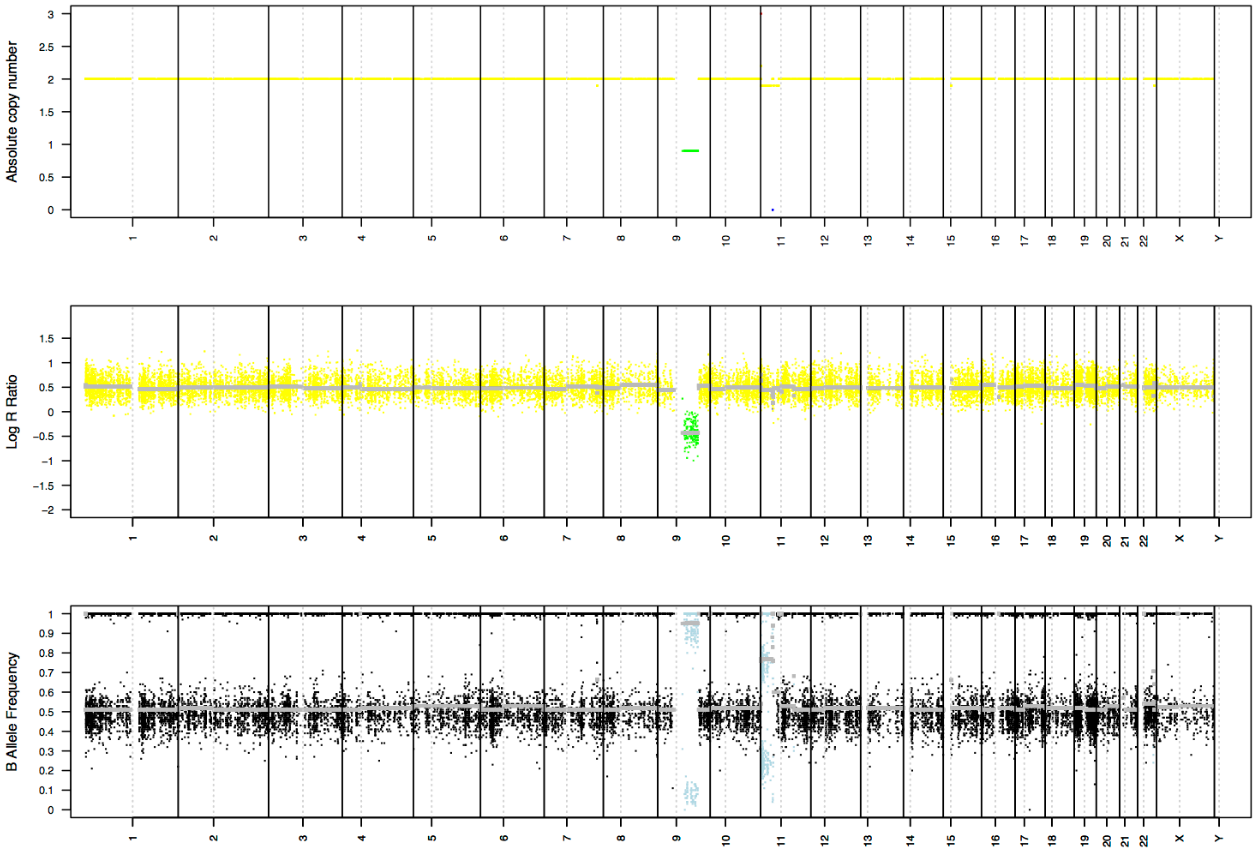

Relapse

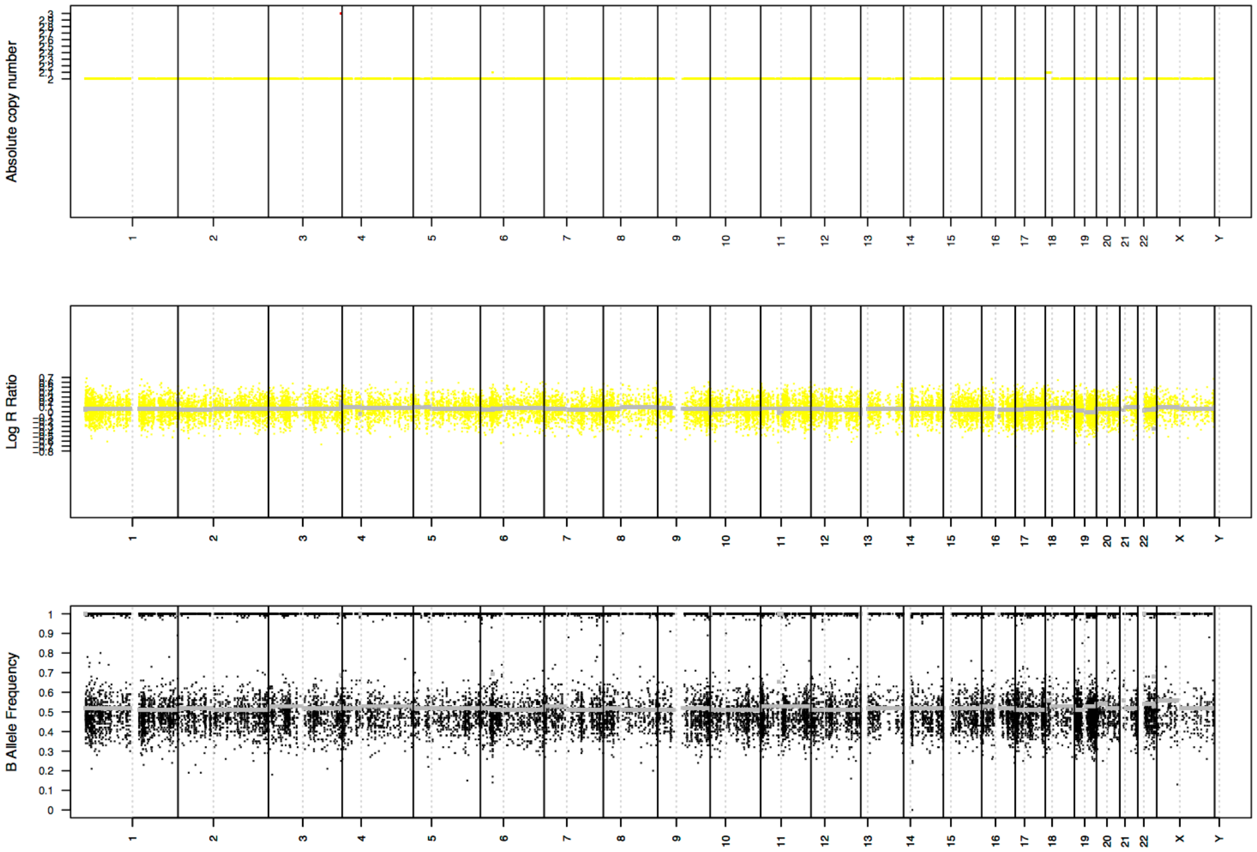

Color code: yellow=normal copy-number, green = deletion, red=gain, lightblue=loss of heterozygosity.

Patient P33

Diagnosis

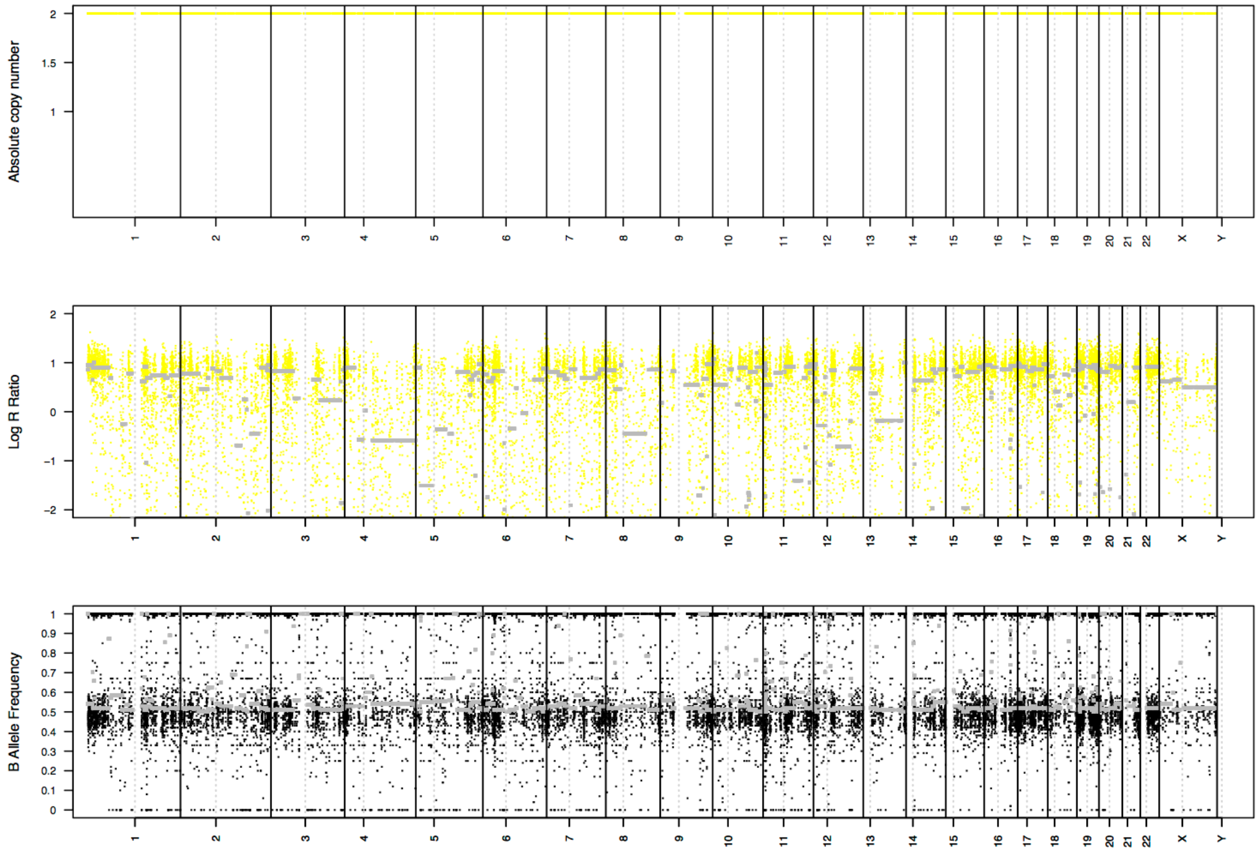

Relapse

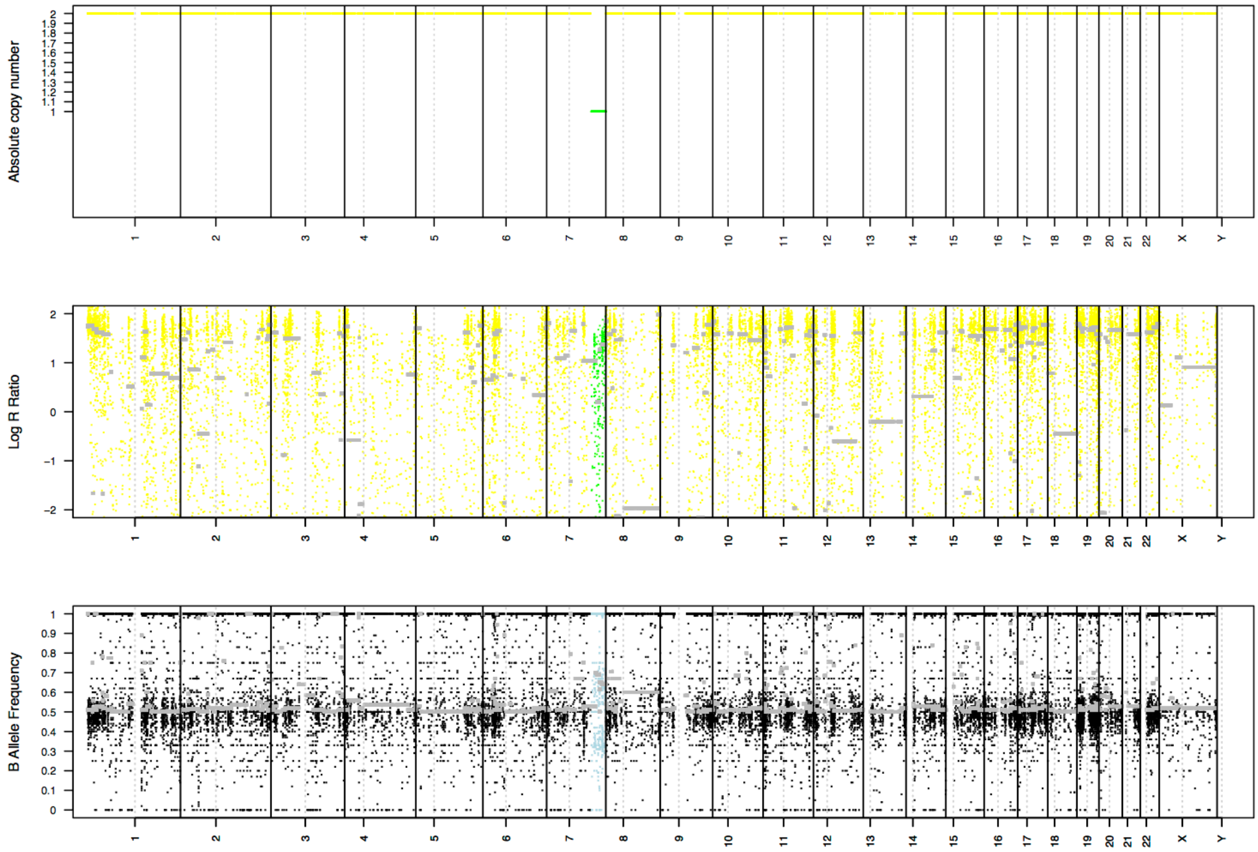

Color code: yellow=normal copy-number, green = deletion, red=gain, lightblue=loss of heterozygosity.

Patient P5

Diagnosis

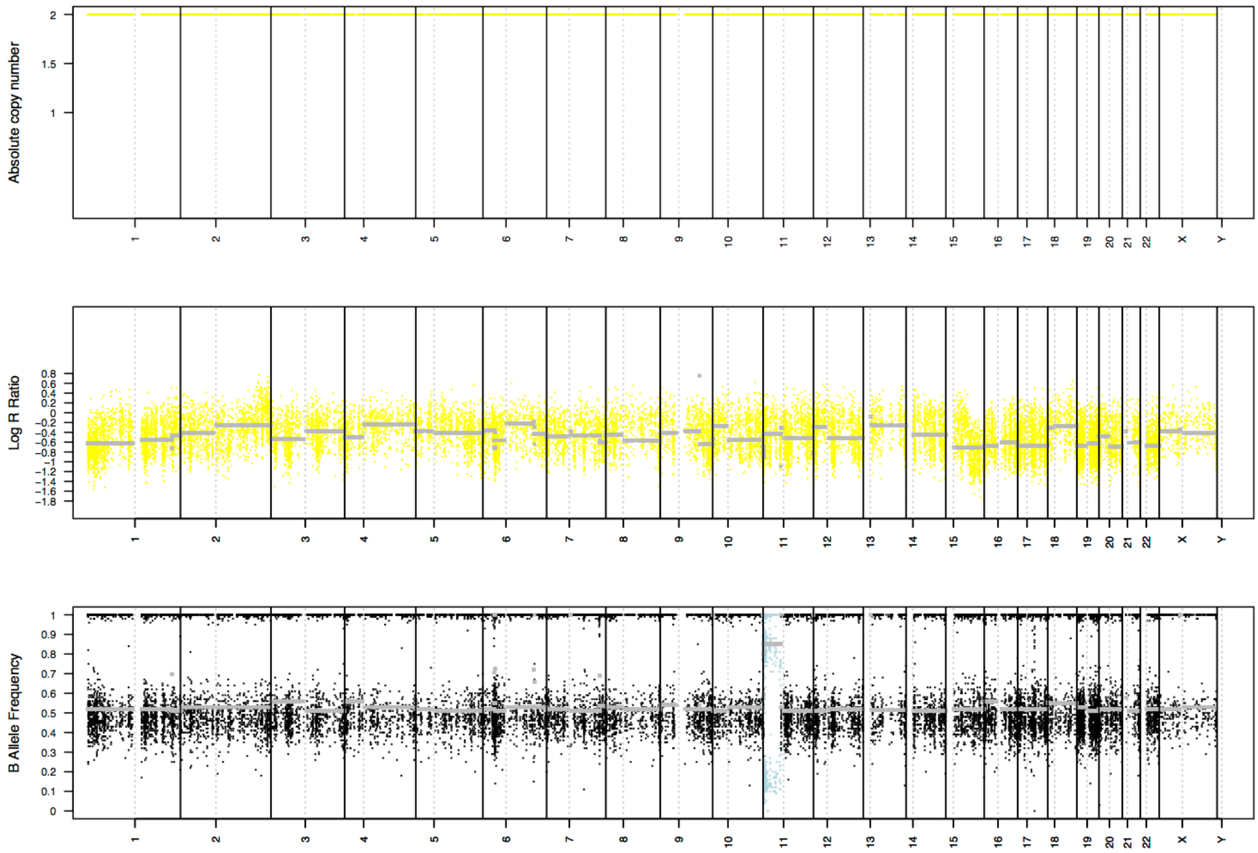

Relapse

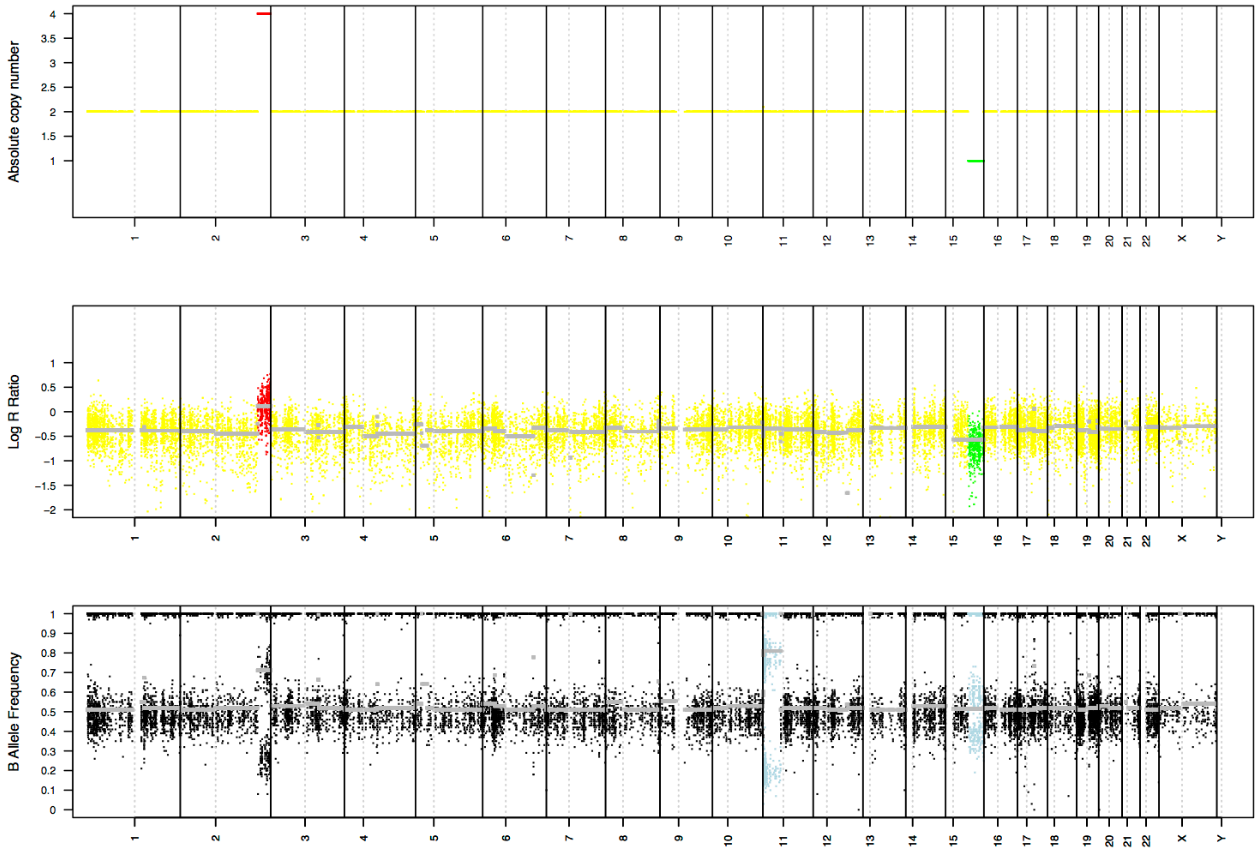

Color code: yellow=normal copy-number, green = deletion, red=gain, lightblue=loss of heterozygosity.

Patient P8

Diagnosis

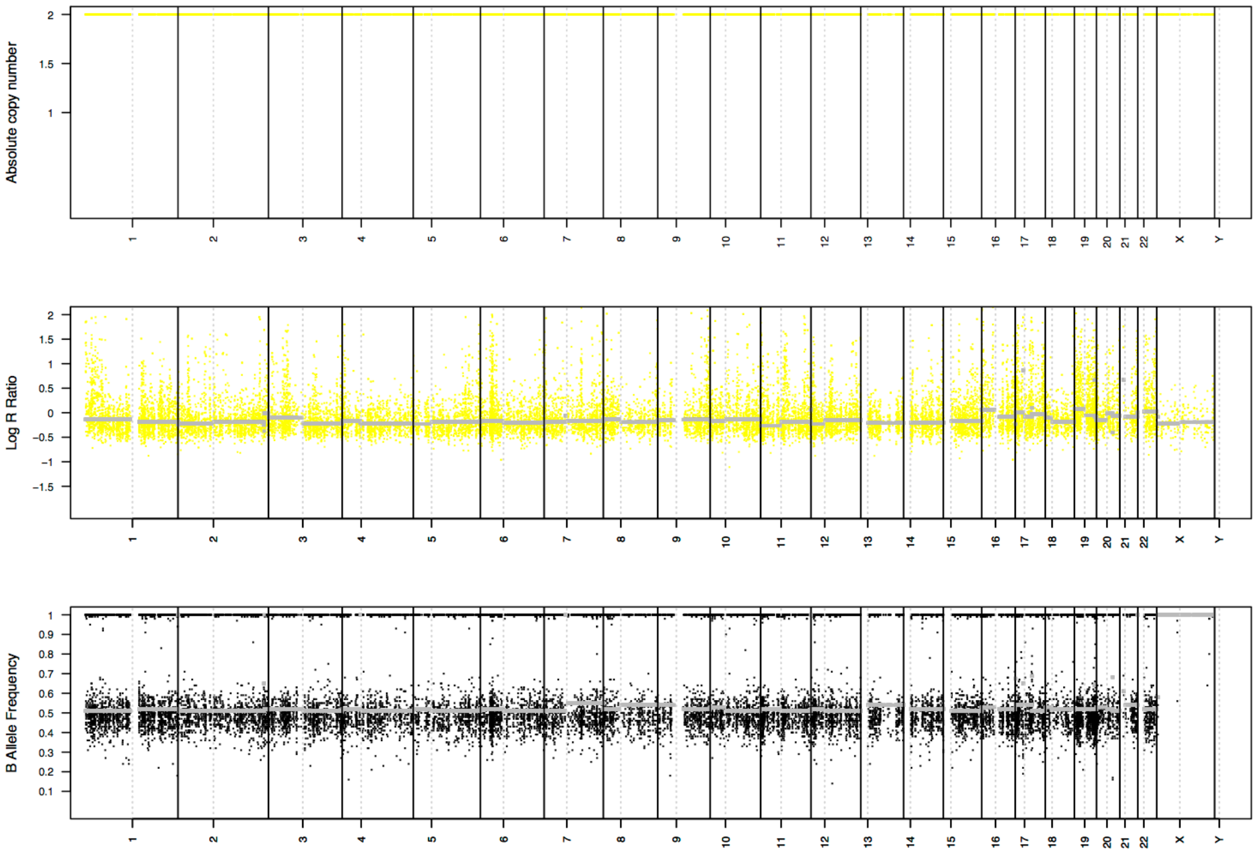

Relapse

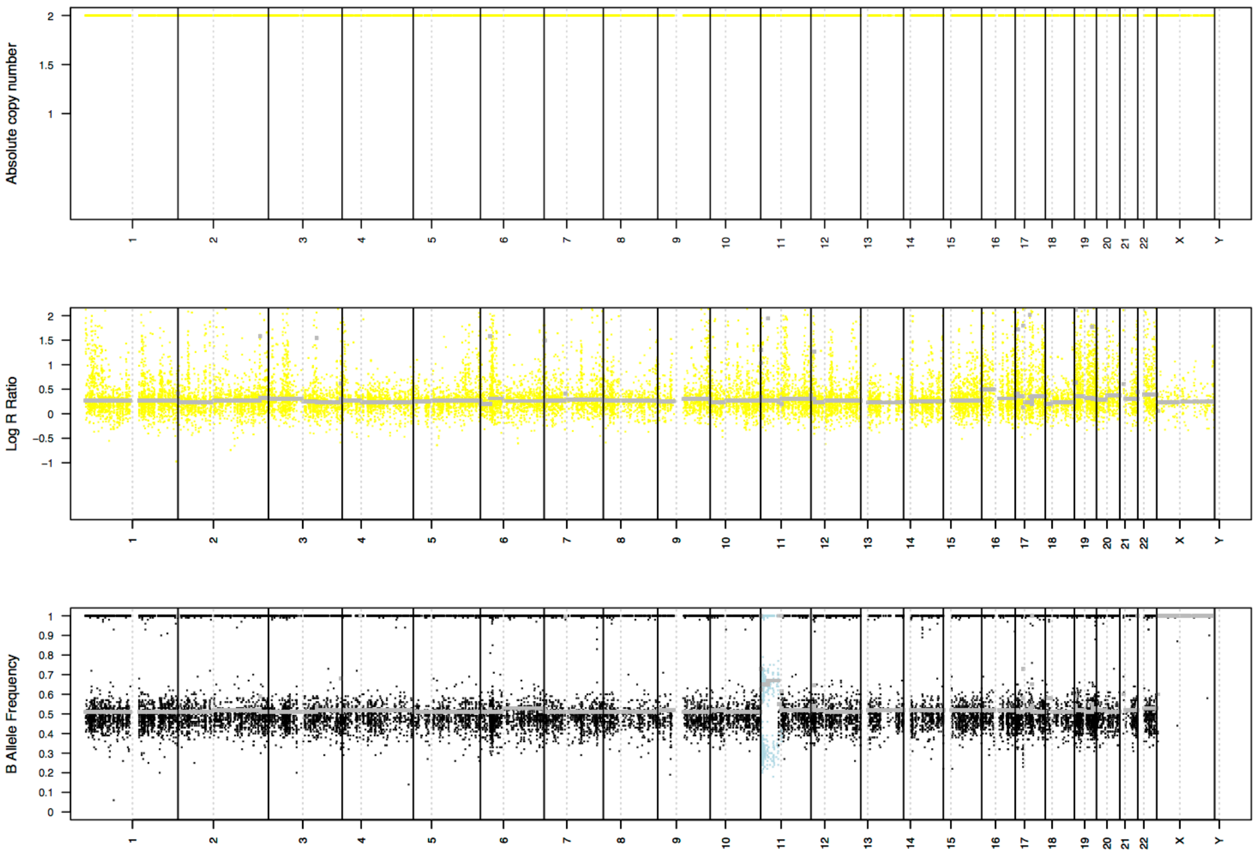

Color code: yellow=normal copy-number, green = deletion, red=gain, lightblue=loss of heterozygosity.

Patient P9

Diagnosis

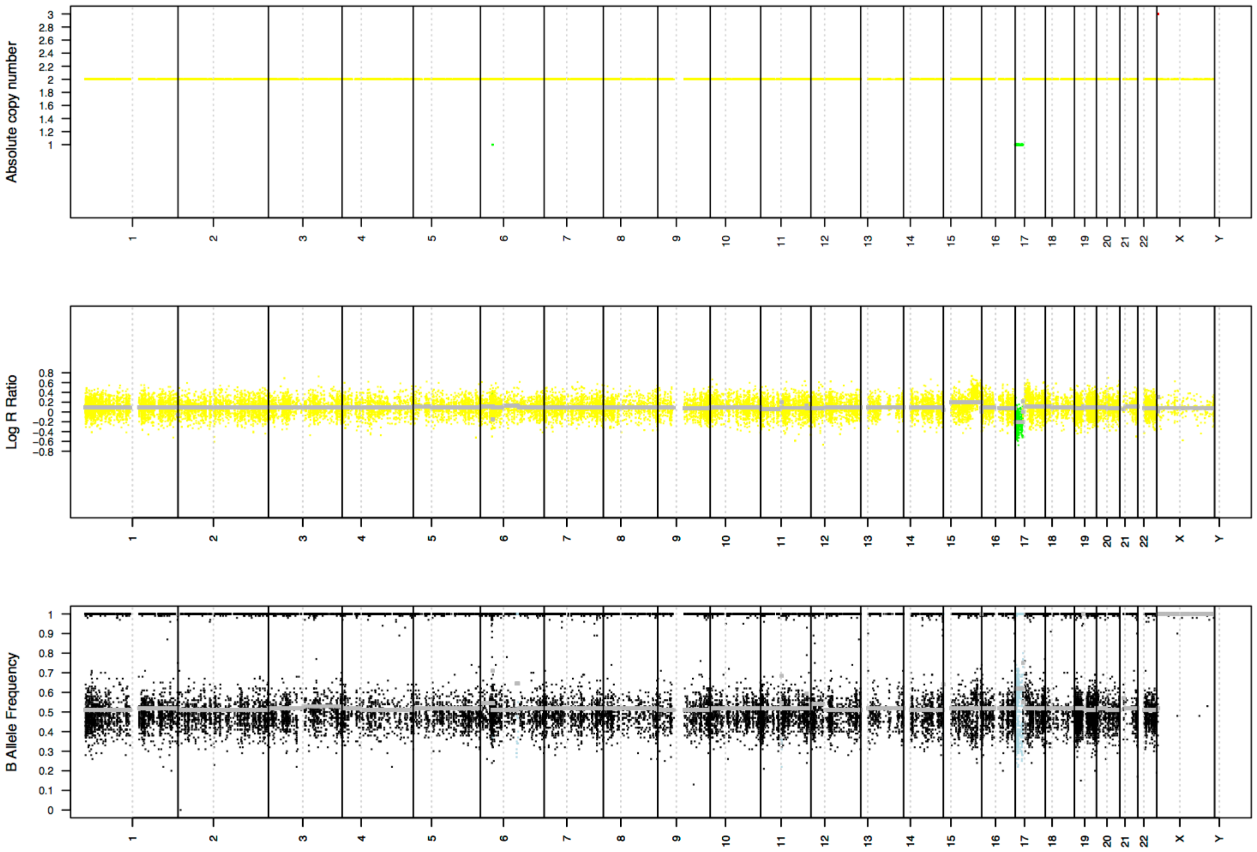

Relapse

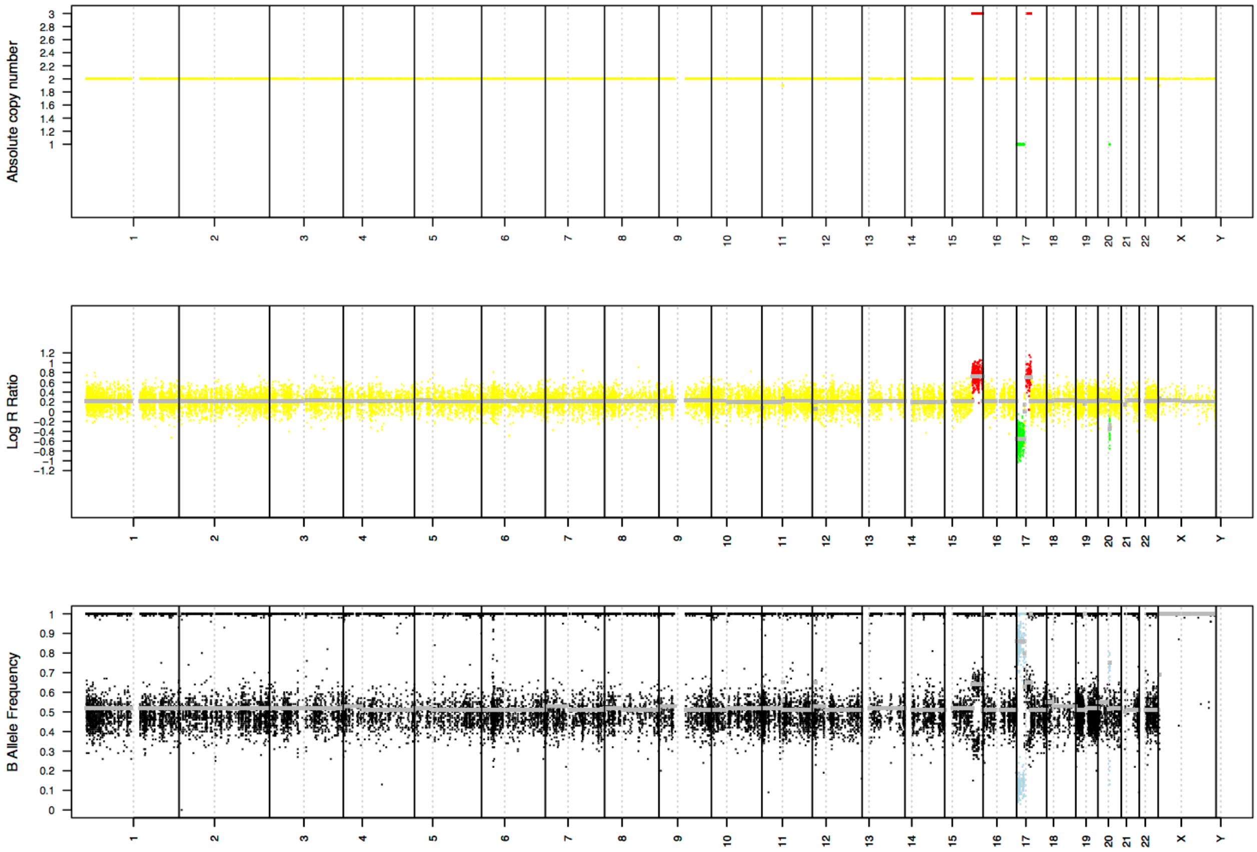

Color code: yellow=normal copy-number, green = deletion, red=gain, lightblue=loss of heterozygosity.

Supplementary Figure 2: summary of the copy number alterations.

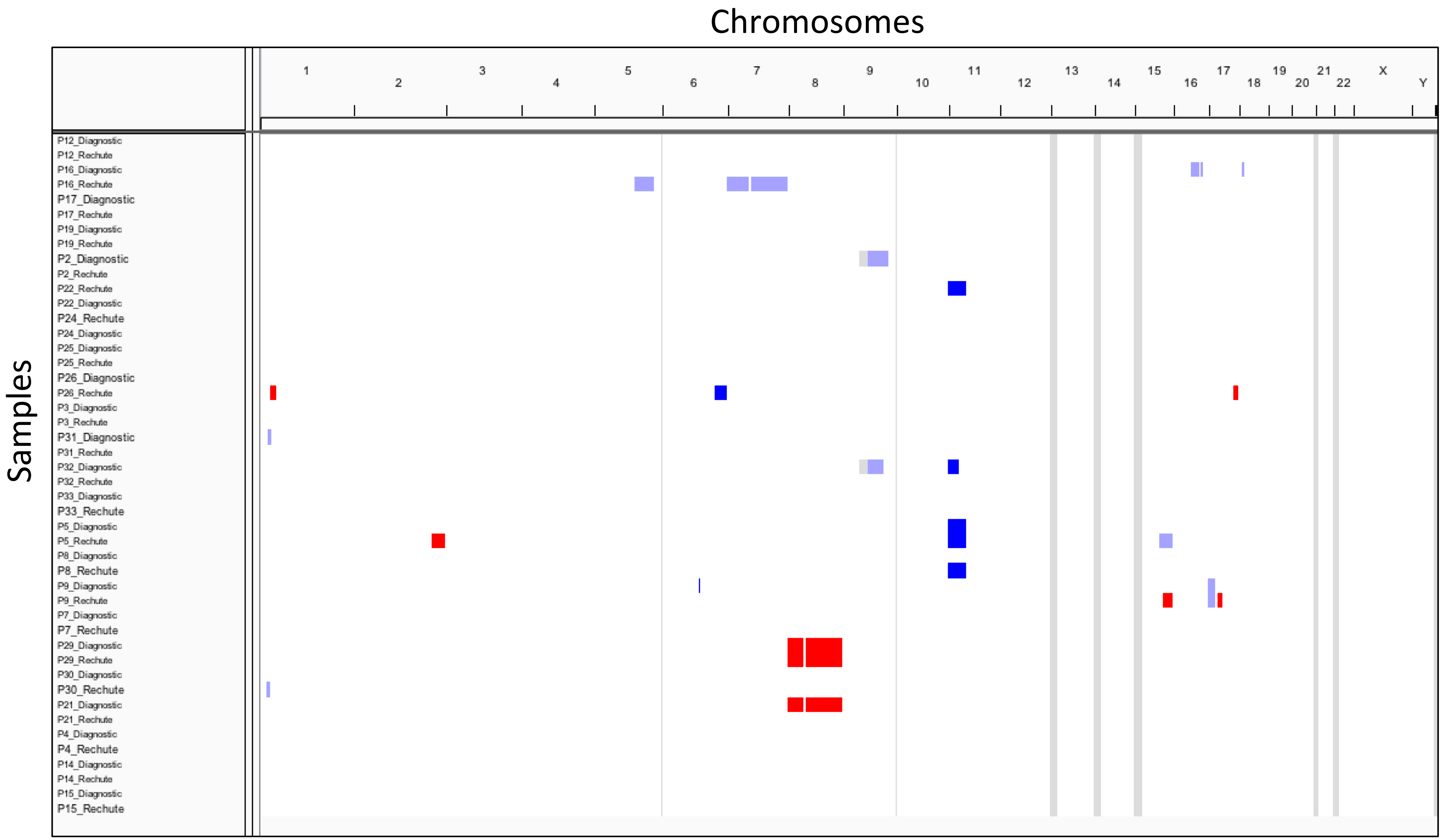

Supplementary Figure 3: Cancer Cell Fraction (CCF) in the 18 patients with available germline DNA. Mutations present at diagnosis and relapse are indicated in black, those solely present at diagnosis in blue, those solely present at relapse in red. Recurrent or driver alterations listed in Supplementary Table 3 are indicated.

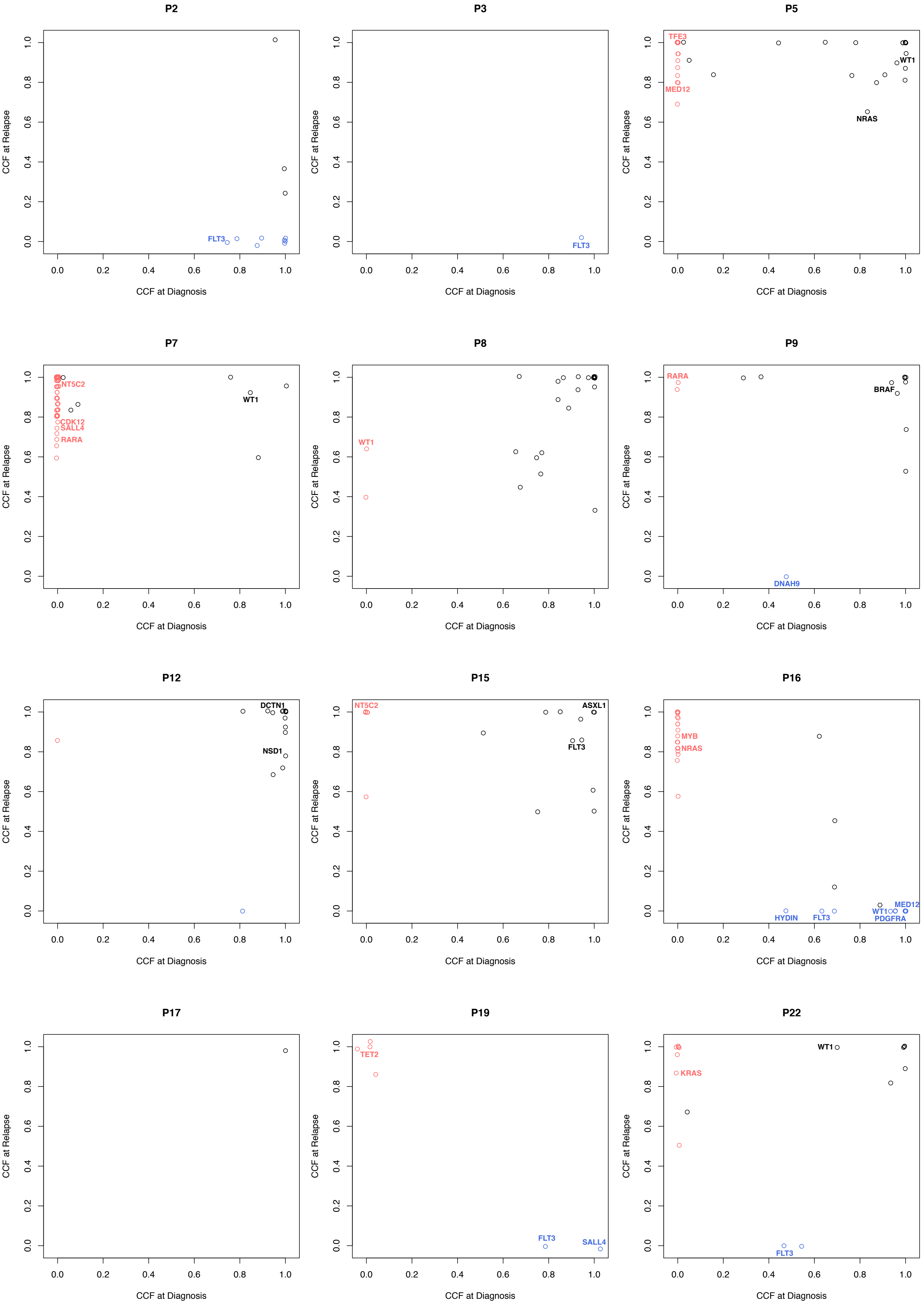
P2
FLT3
P3
FLT3
P5
TFE3
MED12
WT1
NRAS
P7
NT5C2
CDK12
SALL4
RARA
WT1
P8
WT1
P9
RARA
BRAF
DNAH9
P12
DCTN1
NSD1
P15
NT5C2
ASXL1
FLT3
P16
MYB
NRAS
HYDIN
FLT3
WT1
MED12
PDGFRA
P17
P19
TET2
FLT3
SALL4
P22
KRAS
WT1
FLT3
CCF at Relapse
CCF at Diagnosis

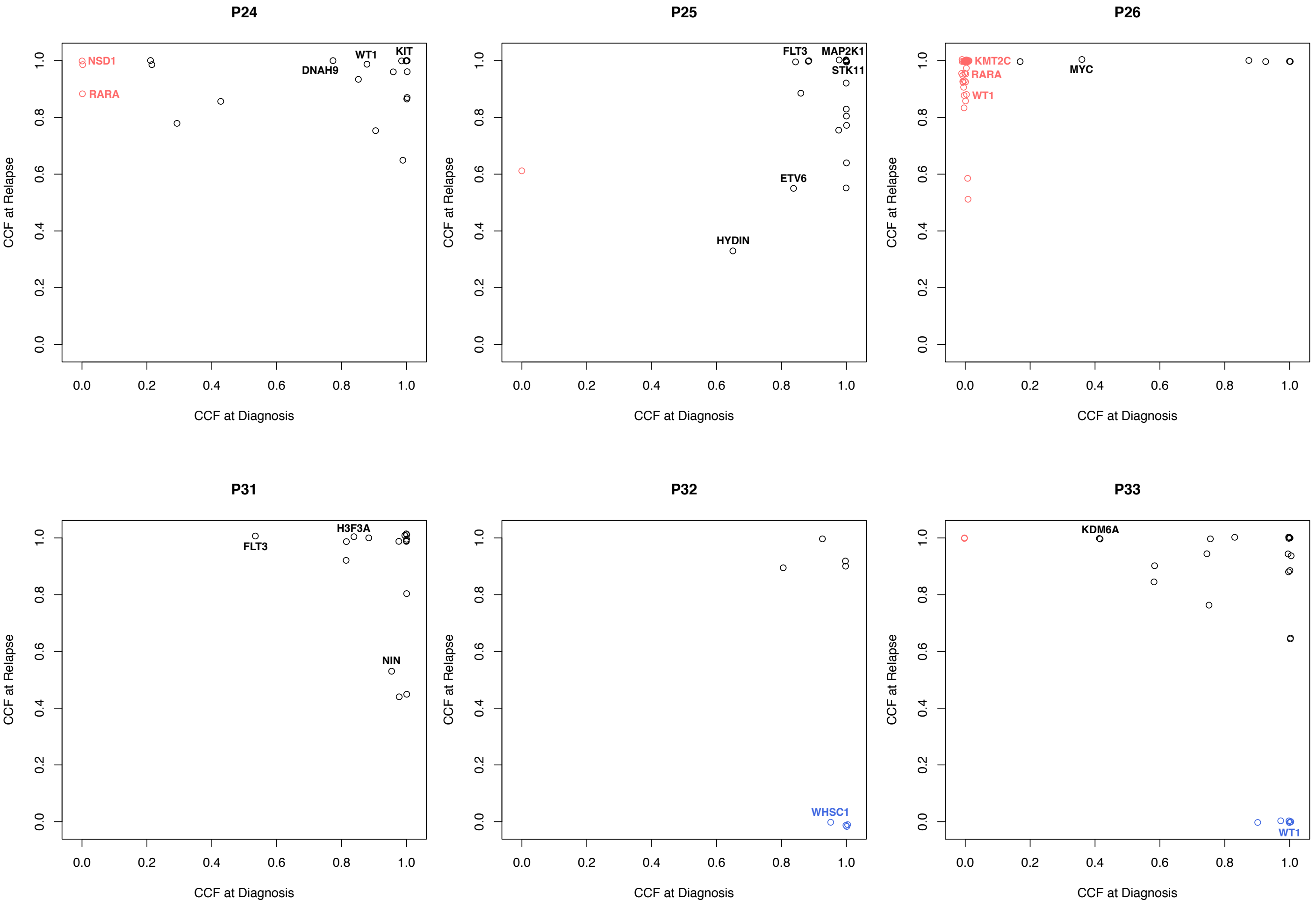
P24
NSD1
RARA
DNAH9
WT1
KIT
CCF at Relapse
CCF at Diagnosis
P25
FLT3
MAP2K1
STK11
ETV6
HYDIN
CCF at Relapse
CCF at Diagnosis
P26
KMT2C
RARA
WT1
MYC
CCF at Relapse
CCF at Diagnosis
P31
H3F3A
FLT3
NIN
CCF at Relapse
CCF at Diagnosis
P32
WHSC1
CCF at Relapse
CCF at Diagnosis
P33
KDM6A
WT1
CCF at Relapse
CCF at Diagnosis

Supplementary Table 1: Available patient features.

UPN	age at diagnosis	M/F	WBC at diagnosis (G/L)	cytological form	time from diagnosis to first relapse (months)
**P2**	64	M	30.2	variant	37
**P3**	41	M	1.5	classical	17
**P4**	43	M	14.9	classical	17
**P5**	56	F	60.5	classical	41
**P7**	2	M	100	variant	27
**P8**	4	M	5	classical	46
**P9**	63	M	5.1	classical	16
**P12**	40	M	3.4	classical	59
**P14**	19	M	2	classical	26
**P15**	56	F	3.2	classical	36
**P16**	62	F	>5	N/A	39
**P17**	58	F	1.1	classical	35
**P19**	57	F	15.9	N/A	54
**P21**	18	F	1.1	classical	35
**P22**	44	M	6	N/A	13
**P24**	55	M	1.4	classical	9
**P25**	47	F	3.2	classical	27
**P26**	38	M	<10	N/A	35
**P29**	64	M	100	variant	28
**P30**	66	M	5.4	N/A	25
**P31**	53	M	1.9	classical	37
**P32**	37	F	8.5	classical	57
**P33**	51	F	N/A	N/A	108

Supplementary Table 2: Comparison of driver gene mutation frequencies in our patient set, Madan and the TCGA AML (M3) and non-AML (non-M3) series

Gene	Primary APL This study	Relpase APL This study	Primary APL Madan *et al.*	Relapse APL Madan *et al.*	TCGA M3*	TCGA non-M3
FLT3	44,4	11,1	32,7	29,9	30,0	27,8
WT1	33,3	38,9	12,1	18,2	5,0	6,1
DNAH9	11,1	5,6	0	0	5,0	1,7
HYDIN	11,1	5,6	0	0	5,0	0,6
NRAS	5,6	11,1	9,1	5,2	0	8,3
NSD1	5,6	11,1	0	1,3	0	0,6
ETV6	5,6	5,6	1,2	3,9	5,0	0,6
KIT	5,6	5,6	0,6	0	0	4,4
MED12	5,6	5,6	NA	NA	0	1,1
SALL4	5,6	5,6	NA	NA	0	0
RARA	0	22,2	0	9,1	0	0
NT5C2	0	11,1	NA	NA	0	0
KRAS	0	5,6	3,6	1,3	0	4,4
TET2	0	5,6	1,2	0	0	9,4
KMT2C	0	5,6	NA	NA	0	0
ARID1A	0	0	4,2	5,2	0	0,6
ARID1B	0	0	3	10,4	0	0
LRP1	0	0	2,4	2,6	0	0
USP9X	0	0	1,8	1,3	5,0	0,6
ABCA7	0	0	1,8	0	0	0
EZH2	0	0	1,2	1,3	0	1,7
CEBPE	0	0	1,2	1,3	0	0,6
CENPF	0	0	1,2	1,3	0	0,6
NUMA1	0	0	1,2	1,3	0	0,6
LRRC4C	0	0	1,2	1,3	0	0
KCNH5	0	0	1,2	0	0	0,6
SETD1B	0	0	1,2	0	0	0
RUNX1	0	0	0,6	6,5	0	10,6
DNMT3A	0	0	0,6	0	0	28,3
TP53	0	0	0	2,6	0	8,9
NPM1	0	0	0	0	5,0	2,8
PTPN11	0	0	0	0	0	30
U2AF1	0	0	0	0	0	5
SMC3	0	0	0	0	0	4,4
SMC1A	0	0	0	0	0	3,9
STAG2	0	0	0	0	0	3,9
PHF6	0	0	0	0	0	3,9
RAD21	0	0	0	0	0	2,8
FAM5C	0	0	NA	NA	5,0	2,2
RUNX1T1	0	0	NA	NA	0	1,1
HNRNPK	0	0	NA	NA	0	1,1
CEBP1	0	0	NA	NA	0	0
IDF1	0	0	NA	NA	0	0
IDF2	0	0	NA	NA	0	0

*M3 group corresponds to APL in the TCGA study, NA: Genes not analyzed bby Madan *et al.* (capture or 398 genes)

Supplementary Table 3: Number of mutations in each patient at different stages of tumor progression

Patient	Number of mutations common to Diagnosis and Relapse	Drivers common to Diagnosis and Relapse	Number of mutations specific to Diagnosis	Drivers specific to Diagnosis	Number of mutations specific to Relapse	Drivers specific to Relapse
P5	21	NRAS, WT1	0		18	TFE3, MED12
P7	7	WT1	0		58	NT5C2, CDK12, RARA, SALL4
P8	25		0		2	WT1
P15	11	FLT3, ASXL1	0		5	NT5C2
P17	1		0		0	
P24	19	WT1, DNAH9, KIT	0		3	RARA, NSD1
P25	20	ETV6, FLT3, MAP2K1, HYDIN, STK11	0		1	
P26	6	MYC	0		36	WT1, RARA, KMT2C
P31	16	H3F3A, FLT3, NIN	0		0	
P9	10	BRAF	1	DNAH9	2	RARA
P12	15	DCTN1, NSD1	1		1	
P16	4		15	WT1, FLT3, HYDIN, PDGFRA, MED12	24	NRAS, MYB
P22	7	WT1	2	FLT3	7	KRAS
P33	18	KDM6A	7	WT1	2	
P2	3		8	FLT3	0	
P3	0		1	FLT3	0	
P19	0		2	FLT3, SALL4	4	TET2
P32	4		4	WHSC1	0	

Supplementary Table 4: Number of mutations in recurrent genes at different stages of tumor progression

Gene	Number of mutations common to Diagnosis and Relapse	Number of mutations specific to Diagnosis	Drivers specific to Relapse
FLT3	4	5	0
WT1	4	2	2
DNAH9	1	1	0
HYDIN	1	1	0
MED12	0	1	1
NRAS	1	0	1
NSD1	1	0	1
SALL4	0	1	1
NT5C2	0	0	3
RARA	0	0	4

Supplementary Table 5: Summary of the detected WT1 alterations at diagnosis or relapse.

Patient	Chromosome aberration at diagnosis	Mutation at diagnosis	Chromosome aberration at relapse	Mutation at relapse	Event acquired at relapse
P5	cnLOH	R458*	cnLOH	R458*	
P7		K400*		K400*	
P8			cnLOH	R242fs	R242fs + cnLOH
P16		D367_R369del		D367_R369del	
P22		Q259*	cnLOH	Q259*	cnLOH
P24		H465N		H465N	
P26				R462W	R462W
P32	cnLOH	hdel			
P33		G447splice			

Supplementary Table 6: Summary of chromosomal alterations.

Patient	Chrom.	Start position	End position	Start cytoband	End cytoband	Type	Common to Diagnosis and Relapse	Specific to Diagnosis	Specific to Relapse
P2	9	66457267	120053776	chr9q13	chr9q33.1	deletion	no	yes	no
P5	11	250877	47603733	chr11p15.5	chr11p11.2	copy-neutral LOH	yes	no	no
P5	2	207998800	242839363	chr2q33.3	chr2q37.3	gain	no	no	yes
P5	15	65621857	102312835	chr15q22.31	chr15q26.3	deletion	no	no	yes
P8	11	293188	47800723	chr11p15.5	chr11p11.2	copy-neutral LOH	no	no	yes
P9	17	6115	21199083	chr17p13.3	chr17p11.2	deletion	yes	no	no
P9	15	74328116	102312835	chr15q24.1	chr15q26.3	gain	no	no	yes
P9	17	25628820	38418766	chr17q11.1	chr17q21.2	gain	no	no	yes
P9	20	31427635	32379263	chr20q11.21	chr20q11.22	deletion	no	no	yes
P16	15	74328116	74753112	chr15q24.1	chr15q24.1	deletion	no	yes	no
P16	16	48122582	77398081	chr16q12.1	chr16q23.1	deletion	no	yes	no
P16	18	10758023	15004191	chr18p11.22	chr18p11.21	deletion	no	yes	no
P16	5	108516629	159835658	chr5q21.3	chr5q33.3	deletion	no	no	yes
P16	7	296739	158930751	chr7p22.3	chr7q36.3	deletion	no	no	yes
P21	8	196274	146228622	chr8p23.3	chr8q24.3	gain	no	yes	no
P22	11	193865	47354905	chr11p15.5	chr11p11.2	copy-neutral LOH	no	no	yes
P26	1	31740706	46870761	chr1p35.2	chr1p33	gain	no	no	yes
P26	6	139183879	170892848	chr6q24.1	chr6q27	deletion	no	no	yes
P26	17	67109894	81050982	chr17q24.3	chr17q25.3	gain	no	no	yes
P29	8	196274	146228622	chr8p23.3	chr8q24.3	gain	yes	no	no
P30	1	20020993	29842261	chr1p36.13	chr1p35.3	deletion	no	no	yes
P31	1	23688933	32557486	chr1p36.12	chr1p35.1	deletion	yes	no	no
P32	9	66457267	108510483	chr9q13	chr9q31.2	deletion	no	yes	no
P32	11	193698	33780163	chr11p15.5	chr11p13	copy-neutral LOH	no	yes	no
P32	11	32124579	32460676	chr11p13	chr11p13	homozygous deletion	no	yes	no
P33	7	131815220	106513011	chr7q32.3	chr7q22.3	deletion	no	no	yes
