# Supplementary material for: Dual origin of relapses in retinoic-acid resistant acute promyelocytic leukemia
Source: Nat Commun. 2018 May 24;9:2047. doi: 10.1038/s41467-018-04384-5 (PMC5967331; doi:10.1038/s41467-018-04384-5)
Supplement: Supplementary file 3 — Description of Additional Supplementary Files [file 41467_2018_4384_MOESM3_ESM.pdf]

Description of Additional Supplementary Files:

Supplementary data 1: List of all somatic mutations detected in the APLs at diagnosis or relapse. Cancer Cell Fractions (CCF) are indicated. Alterations are ordered by patient.

Supplementary data 2: List of mutated genes highlighted in the clonal evolution schemes. These include all genes altered at least twice in Supplementary Table 2, as well as any cancer-associated genes from either the Cancer Genome Census or AML-associated genes. Alterations of all these genes were controlled by visual inspection of the primary sequencing data. Alterations are ordered by genes.
